# Surgical procedures in the pilonidal sinus disease: a systematic review and network meta-analysis

**DOI:** 10.1038/s41598-020-70641-7

**Published:** 2020-08-13

**Authors:** Siwei Bi, Kaibo Sun, Shanshan Chen, Jun Gu

**Affiliations:** 1 Department of Burn and Plastic Surgery, West China Hospital, Sichuan University, Chengdu, 610041 Sichuan People’s Republic of China; 2 West China School of Medicine, Sichuan University, Chengdu, 610041 Sichuan People’s Republic of China; 3 Department of Cardiovascular Surgery, West China Hospital, Sichuan University, Chengdu, 610041 Sichuan People’s Republic of China

**Keywords:** Diseases, Outcomes research

## Abstract

The most appropriate surgical treatment for pilonidal sinus disease (PSD) is still in dispute. This study aims to comprehensively compare the outcomes of surgical interventions using network meta-analysis. Randomized controlled trial studies were searched systematically to identify all eligible studies in multiple databases and previous publications and Bayesian network meta-analysis was performed. Our primary outcome was the recurrence rate. Differences in the findings of the studies were explored in meta regressions and sensitivity analyses. The risk of bias of each study was assessed using the Cochrane risk of bias tool. Confidence in evidence was assessed using CINeMA (Confidence in Network Meta-Analysis). A total of 39 studies and 5,061 patients were identified and the most common surgical intervention was the Limberg flap. In network meta-analysis, modified Limberg flap and off-midline closure were associated with the lowest recurrence rate. However, the Karydakis flap was associated with shorter operation time by several minutes compared with other interventions and few significant results were found in other outcomes. Modified Limberg flap and off-midline closure provided relatively low recurrence and complications rates. Therefore, they could be two promising surgical interventions for PSD patients.

## Introduction

Pilonidal sinus disease (PSD) is a tract or cavity of the sacrococcygeal region characterized by repeated infection and chronic inflammation. PSD reportedly has an incidence rate of 26 per 100,000 people and predominately affects young males^1^. Risk factors include poor hygiene, obesity, and unhealthy behavior such as prolonged sitting^2^. Presenting symptoms vary from acute to chronic, with one or more non-inflamed pits in the natal cleft as the most common manifestation^1,3^. Although it is a benign disorder, it can be fairly painful and cause absence from work and school that greatly affects the quality of life. Moreover, it has a high risk of recurrence and brings many complications such as infection, chronic nonhealing wounds, and even squamous cell carcinoma within sinus tracts. Due to the constant exposure to pathogenic microbes, the recurrent abscesses can usually last for many years^3–5^. Therefore, the recurrence rate has become an important parameter for evaluating the treatments’ effects.

Clinicians nowadays are facing various surgical choices in treatments of PSD except excision and primary closure (PC), e.g., the off-midline approach including the Limberg flap (LF) and Karydakis flap (KF). LF is considered to be an effective treatment because of its low recurrence and complication rate in addition to a short healing time and hospital stay^6^. Meanwhile, KF is associated with significantly lower rates of complications and recurrence compared with excision only^7^. Some reports showed that the LF had a lower rate of postoperative complications and greater cosmetic satisfaction than KF^8,9^, while others reported that there are no significant differences between LF and KF^10,11^. Besides, minimally invasive techniques (MIT) such as phenol injection, unroofing and marsupialization, video-assisted ablation^12^ and endoscopic sinus treatment^13^ are also emerging as alternative procedures.

Network meta-analysis (NMA) facilitates the comparisons between collected interventions and analyzes the pooled data by connecting a network of evidence. It can also give a relative ranking of treatments included^14^. The obvious advantage is that all comparisons can be evaluated simultaneously because the indirect comparisons gaps can be filled. Additionally, with the combination of direct and indirect evidences, a greater statistical power and precision can be obtained^15^.

Literature search depicts, few previous pairwise meta-analysis comprehensively integrated all the surgical techniques and measured their effect in a wide range of outcomes. The aim of this study is to perform an NMA with randomized, controlled trials (RCTs) from present publications focusing on the comparisons of surgical interventions for PSD and to better inform clinical practice.

## Results

### Search results, study characteristics

**Figure 1:**
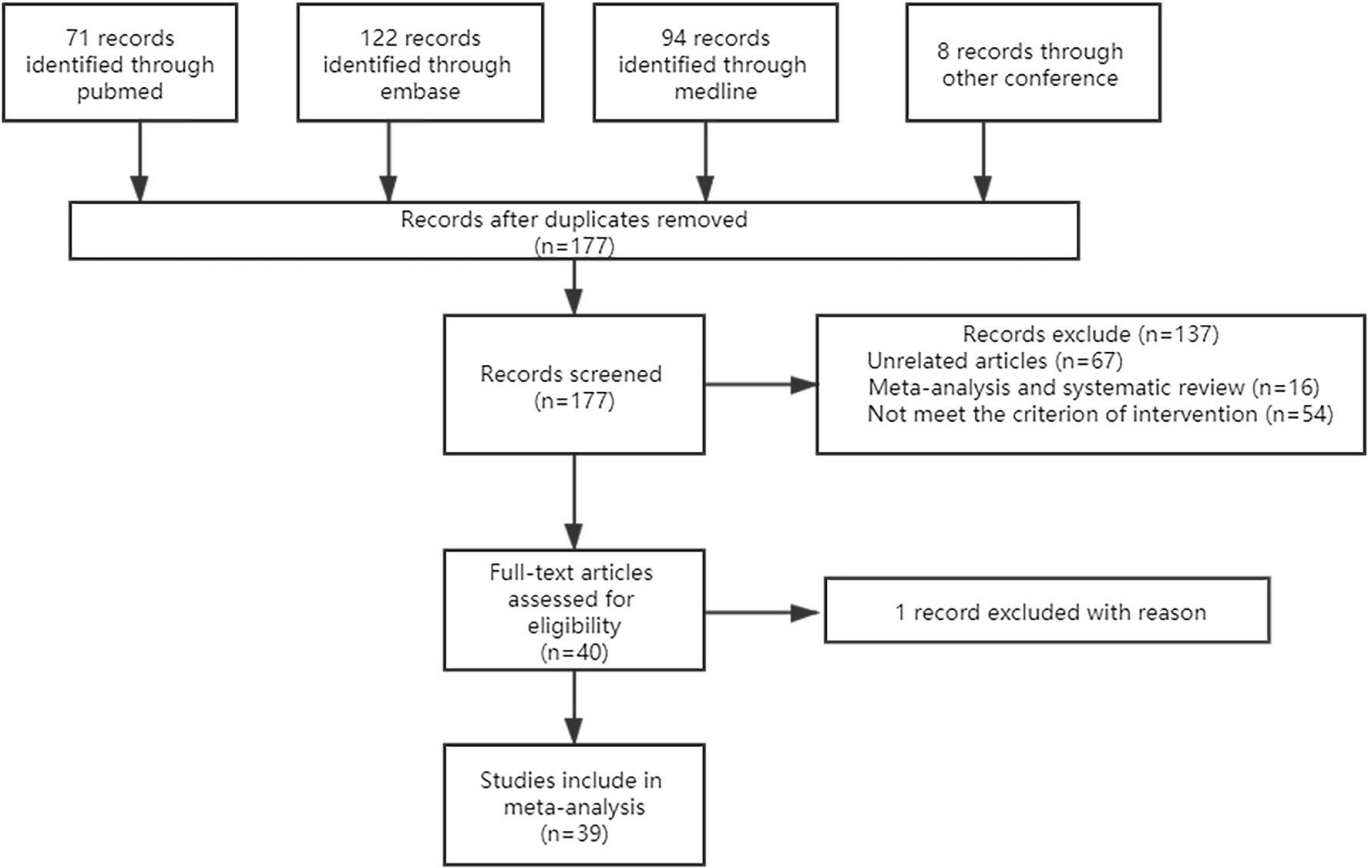
Flowchart for searching and identifying eligible studies.

**Figure 2:**
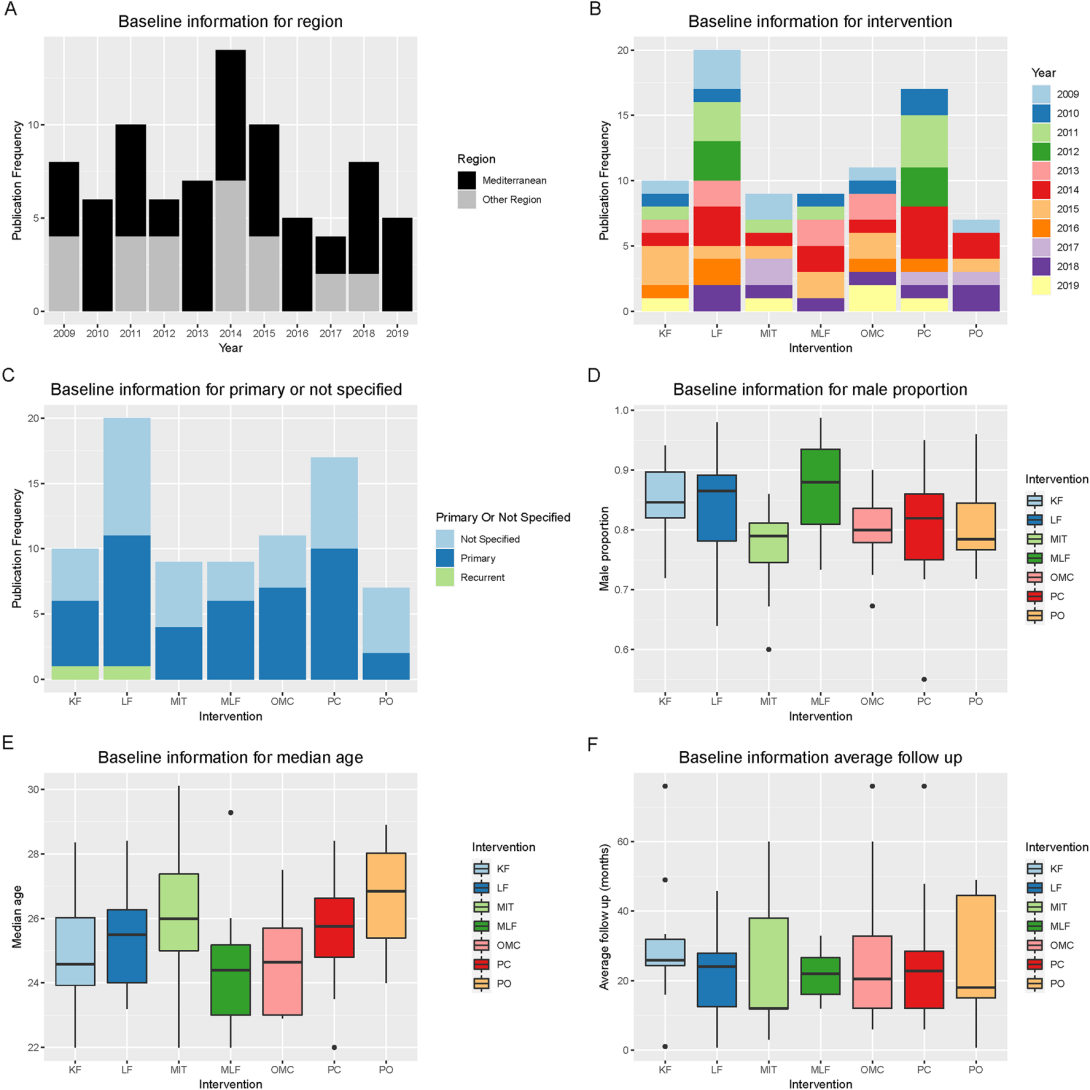
The transitivity analysis of potential effect modifiers (**A**). The geography information (Mediterranean or other regions) for the included trials. (**B**) The frequency of each surgical intervention published in clinical trials. (**C**) The patient information (primary, recurrent or not specified) for each intervention. (**D**–**F**) The male proportion, median age, and average follow-up duration (month). *KF* Karydakis flap, *PO* primary open, *PC* primary closure, *LF* Limberg flap, *MLF* modified Limberg flap, *OMC* off-midline closure, *MIT* minimal invasive technique.

The flowchart for the study selection process is shown (Fig. 1). A total of 295 studies was selected from the aforementioned databases for further screening. We excluded 177 duplicated articles, meta-analysis and systematic review (n = 16), and 137 other articles because of irrelevant topics (n = 67). However, Milone^12,16^ et al. published two papers reporting the same clinical trial in 2020 and 2016. We only included the one in 2020 to gain results from a longer follow-up duration. In the end, a total of 39 controlled studies with 5,061 participants were included for this meta-analysis^7,9,16–52^. The detailed classification and definition of treatments are documented in Supplementary Table S1. The transitivity of potential effect modifiers is illustrated. (Fig. 2) Notably, the majority population of the present study were adult males with an average follow-up duration of 24 months. The most frequent intervention used in surgical practice was LF. The number of studies published in the Mediterranean outnumbered the other regions (e.g., Pakistan, Switzerland, Netherlands, etc.) in 6 years. The LF interventions gained the most attention which was selected as an intervention for comparison in 20 studies. There are more studies that not specifying enrolled patients were primary or not than the ones that clarified. Only the study conducted by Bali^53^ et al. specifically included recurrent PSD patients. There were three outliers in male proportion and five in average follow-up duration. The detailed information was tabulated in Supplementary Table 2. The enhanced funnel plots were illustrated for potential publication bias detection and most of the comparisons in our study showed little bias (Supplementary Figs. S1–S7).

### Study quality assessment and grading the evidence of the network meta-analysis using CINeMA

**Figure 3:**
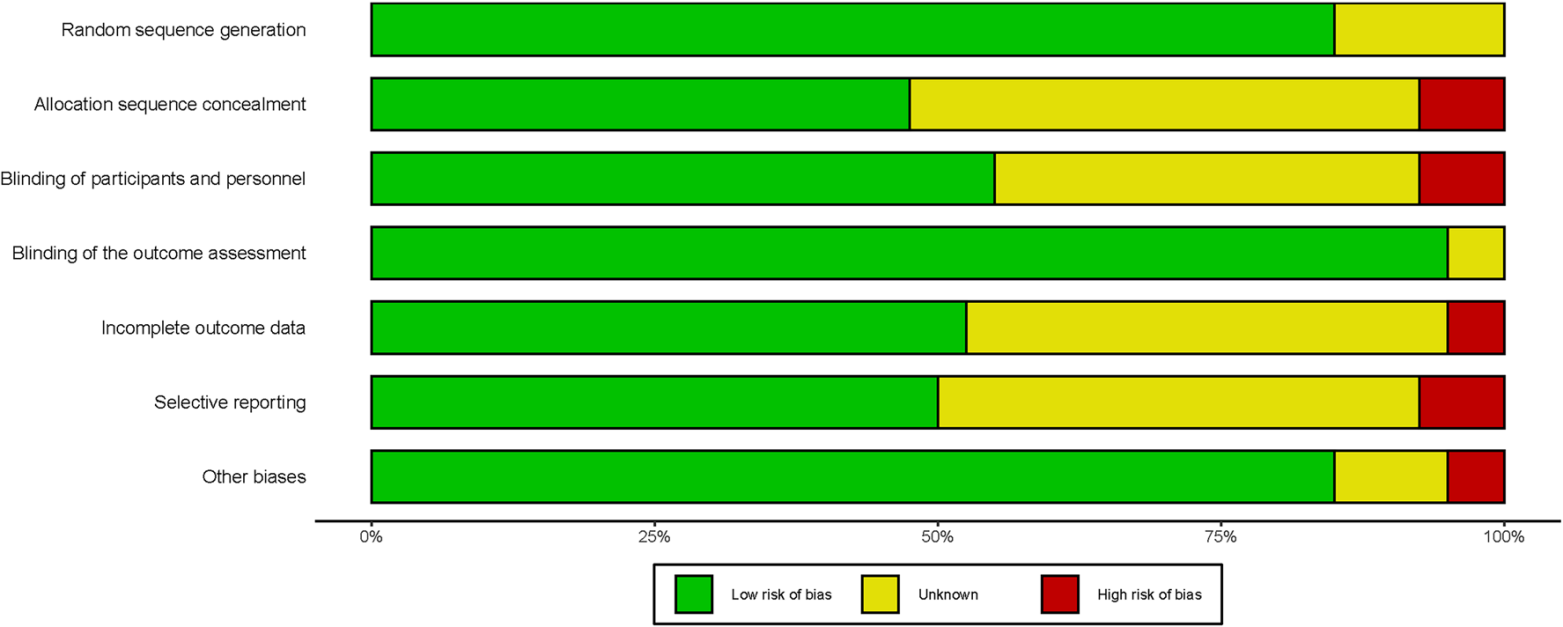
Summary of risk of bias of the included randomized controlled trials.

The summary risk of bias assessment of the 39 included studies was illustrated in Fig. 3. The authors showed the results of each quality item as percentages across studies. Most of the studies are with low risk of bias in all the items. Three studies showed a high risk of bias in allocation sequence concealment, blinding of participants and personnel and selective reporting. Two studies showed incomplete outcome data and other biases. Our judgments described below are based on the recommendations of the online documentation of CINeMA (https://cinema.ispm.ch/#doc). The results were summarized in Table 1 and showed the majority of comparisons were graded as moderate confidence. The most frequent downgrading reason is imprecision.

**Table 1:** Summary meta-analysis results for recurrence and infection.

Outcome	Comparison	Sample size	Number of studies	Omited study	Paiwise meta-analysis				Network meta-analysis			Node splitting (p-Value)	Nature of evidence	CINeMA level	Downgraded reason
					RR/SMD	95% CI		I^2^	RR/SMD	95% CI					
Recurrence	PO vs. LF	142	2	–	1.28	0.49	3.32	75	–	–	–	–	–	–	–
	PO vs. MIT	220	2	–	1.19	0.64	2.2	78	–	–	–	–	–	–	–
	PC vs. KF	225	2	–	1.5	0.43	5.22	33	1.54	0.57	4.39	0.9988	Mixed	Moderate	Heterogeneity
	PC vs. LF	957	8	–	**5.39**	**2.84**	**10.23**	30	1.83	0.87	3.84	0.0607	Mixed	Low	Heterogeneity, incoherence
	PC vs. MLF	180	2	–	4	0.88	18.26	0	2.76	0.99	7.77	0.8402	Mixed	Moderate	Heterogeneity
	PC vs. OMC	374	3	–	0.97	0.47	2.02	0	**2.73**	**1.11**	**7.66**	**0.0129**	Mixed	Low	Heterogeneity, incoherence
	PC vs. MIT	208	3	–	5.36	0.7	41.23	0	1.35	0.35	4.76	0.99705	Mixed	Moderate	Heterogeneity
	KF vs. LF	849	5	–	1.14	0.66	1.96	25	1.19	0.46	2.9	0.98485	Mixed	Moderate	Imprecision
	KF vs. MLF	719	4	Sit, M	1.58	0.08	30.11	56	1.79	0.56	5.72	**0.0147**	Mixed	Moderate	Heterogeneity
	KF vs. OMC	120	1	–	–	–	–	–	1.76	0.55	6.23	0.3072	Mixed	Moderate	Imprecision
	KF vs. MIT	–	–	–	–	–	–	–	0.89	0.17	3.89	–	Indirect	Low	Imprecision,incoherence
	LF vs. MLF	492	2	–	**3.7**	**1.2**	**11.45**	0	1.51	0.53	4.39	**0.0179**	Mixed	Low	Imprecision,incoherence
	LF vs.OMC	203	2	–	0.98	0.15	6.46	0	1.49	0.55	4.5	0.51435	Mixed	Moderate	Imprecision
	LF vs. MIT	140	1		–	–	–	–	0.74	0.17	2.95	–	Mixed	Moderate	Imprecision
	MLF vs. OMC	409	3	–	2.19	0.78	6.12	0	0.99	0.36	3.07	**0.0114**	Mixed	Low	Imprecision,incoherence
	MLF vs. MIT	–	–		–	–	–	–	0.49	0.1	2.14	–	Indirect	Low	Imprecision,incoherence
	OMC vs. MIT	191	2	–	0.88	0.49	1.55	0	0.5	0.12	1.62	0.9983	Mixed	Moderate	Imprecision
Infection	PO vs. PC	40	1		–	–	–	–	0.36	0.09	1.31	0.2863	Mixed	Moderate	Heterogeneity
	PO vs. KF	321	1		–	–	–	–	0.38	0.09	1.43	–	Mixed	Moderate	Imprecision
	PO vs. LF	246	4	–	0.69	0.31	1.52	19	0.51	0.14	1.56	–	Mixed	Moderate	Imprecision
	PO vs. MLF	–	–		–	–	–	–	0.54	0.12	2.15	–	Indirect	Moderate	Imprecision
	PO vs. OMC	–	–		0.58	0.24	1.4	0	0.59	0.14	2.6	–	Indirect	Moderate	Imprecision
	PC vs. KF	100	1		–	–	–	–	1.04	0.39	2.66	0.679	Mixed	Moderate	Imprecision
	PC vs. LF	853	8	–	**2.94**	**1.59**	**5.44**	0	1.4	0.69	2.73	0.6293	Mixed	Low	Heterogeneity, incoherence
	PC vs. MLF	180	2	–	**3.5**	**1.21**	**10.15**	0	1.5	0.58	3.64	0.25695	Mixed	Moderate	Imprecision
	PC vs. OMC	235	2	–	0.58	0.24	1.4	35	1.63	0.64	4.44	**0.0415**	Mixed	Low	Imprecision,incoherence
	KF vs. LF	949	6	–	1.41	0.89	2.24	44	1.35	0.62	2.91	0.70835	Mixed	Moderate	Imprecision
	KF vs. MLF	582	3	–	1.99	0.94	4.23	25	1.44	0.55	3.7	0.3267	Mixed	Moderate	Imprecision
	KF vs. OMC	–	–	–	–	–	–	–	1.57	0.54	5.1	–	Indirect	Moderate	Imprecision
	LF vs. MLF	492	2	–	1.94	0.79	4.79	82	1.07	0.45	2.54	0.13775	Mixed	Moderate	Imprecision
	LF vs.OMC	203	2	–	0.68	0.31	1.53	47	1.16	0.48	3.22	0.52075	Mixed	Moderate	Imprecision
	MLF vs. OMC	409	3	–	0.3	0.09	1.07	0	1.09	0.42	3.19	**0.0033**	Mixed	Low	Imprecision,incoherence

The statistically significant results were shown in bold font.

*KF* Karydakis flap, *PC* primary closure, *LF* Limberg flap, *MLF* modified Limberg flap, *OMC* off-midline closure, *PO* primary open, *MIT* minimum invasive technique, *RR* risk ratio, *SMD* standardized mean difference, *CI* confidential interval, *CINeMA* Confidence in Network Meta-Analysis; Evidence were downgraded from high level of evidence by reasons including: imprecision, incoherence, heterogeneity, reporting bias, within-study bias, indirectness.

### Results for recurrence rate

**Figure 4:**
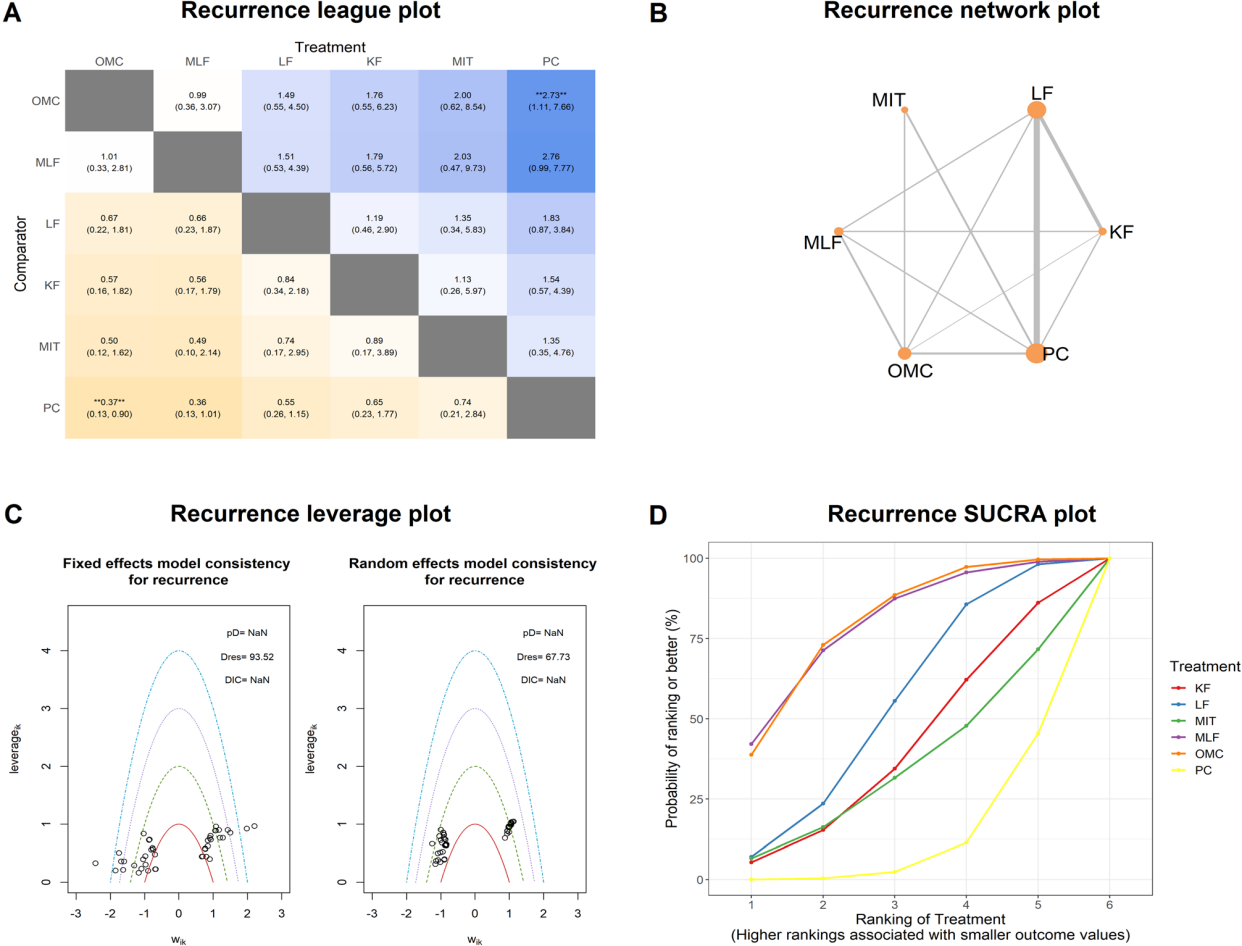
Network analysis results for recurrence. (**A**) The league plot for surgical interventions. The number in each cell represents the comparison between the name of column versus the name of row. Results with statistically significant are annotated with an asterisk. (**B**) The network plot showing the interventions included in the network analysis. Size of nodes represents the sample size of each intervention; edges are the frequency of comparison. (**C**) The leverage plots showing the goodness of random and fixed effects. The model with fewer outliers would be preferred. *Dres* The posterior mean of the residual deviance. *pD* The effective number of parameters, calculated as the sum of the leverages. *DIC* deviance information criterion. (**D**) The surface under the cumulative ranking curve (SUCRA) plot. *KF* Karydakis flap, *PC* primary closure, *LF* Limberg flap, *MLF* modified Limberg flap, *OMC* off-midline closure, *MIT* minimal invasive technique.

**Figure 5:**
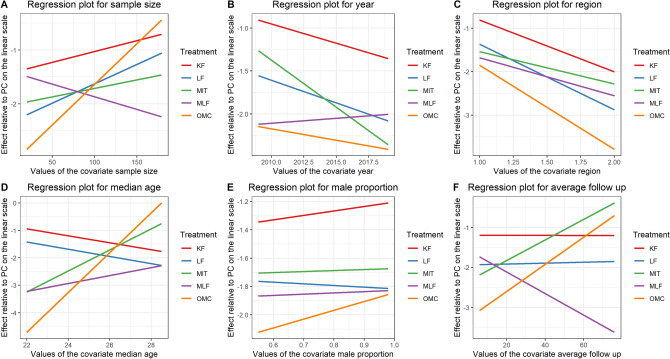
Meta-regression results for recurrence rate adjusted by (**A**) sample size, (**B**) year, (**C**) region, (1 for the Mediterranean area and 2 for the other regions) (**D**) median age, (**E**) male proportion, (**F**) average follow-up. All regressions were plotted as effect relative to primary closure (PC) on the linear scale.

In the pairwise meta-analysis, PC versus LF and LF versus modified Limberg flap (MLF) showed significant results. (RR = 5.39, 95% CI 2.84, 10.23; RR = 3.7, 95% CI 1.2, 11.45, respectively, Table 1). Three heterogeneous comparisons and 3 single study comparisons were excluded before we performed the network meta-analysis. We chose the random model effect and included 3,661 patients, 27 studies and 6 interventions (Fig. 4). Four out of 12 comparisons (MLF versus KF, MLF versus LF, other midline closure (OMC) versus MLF, PC versus OMC) were statistically inconsistent (Table 1). There was a significant increase in the recurrence rate when comparing PC with OMC in network meta-analysis (RR = 2.73, 95% CI 1.11, 7.66, Fig. 4) but not in pairwise results. As shown in the SUCRA plot, the PC was the one with the lowest rankings with the highest recurrence. OMC and MLF presented similar results in the recurrence rate. The meta-regression results were illustrated in Fig. 5. OMC rose the recurrence rate with the increase of sample size, median age and average follow-up. (Fig. 5A,D,F) Interestingly, MLF showed decreased recurrence incidence as the prolongation of average follow-up time. (Fig. 5F) The recurrence rate decreased from the Mediterranean area to the other areas consistently with all interventions. (Fig. 5C).

### Results for infection

**Figure 6:**
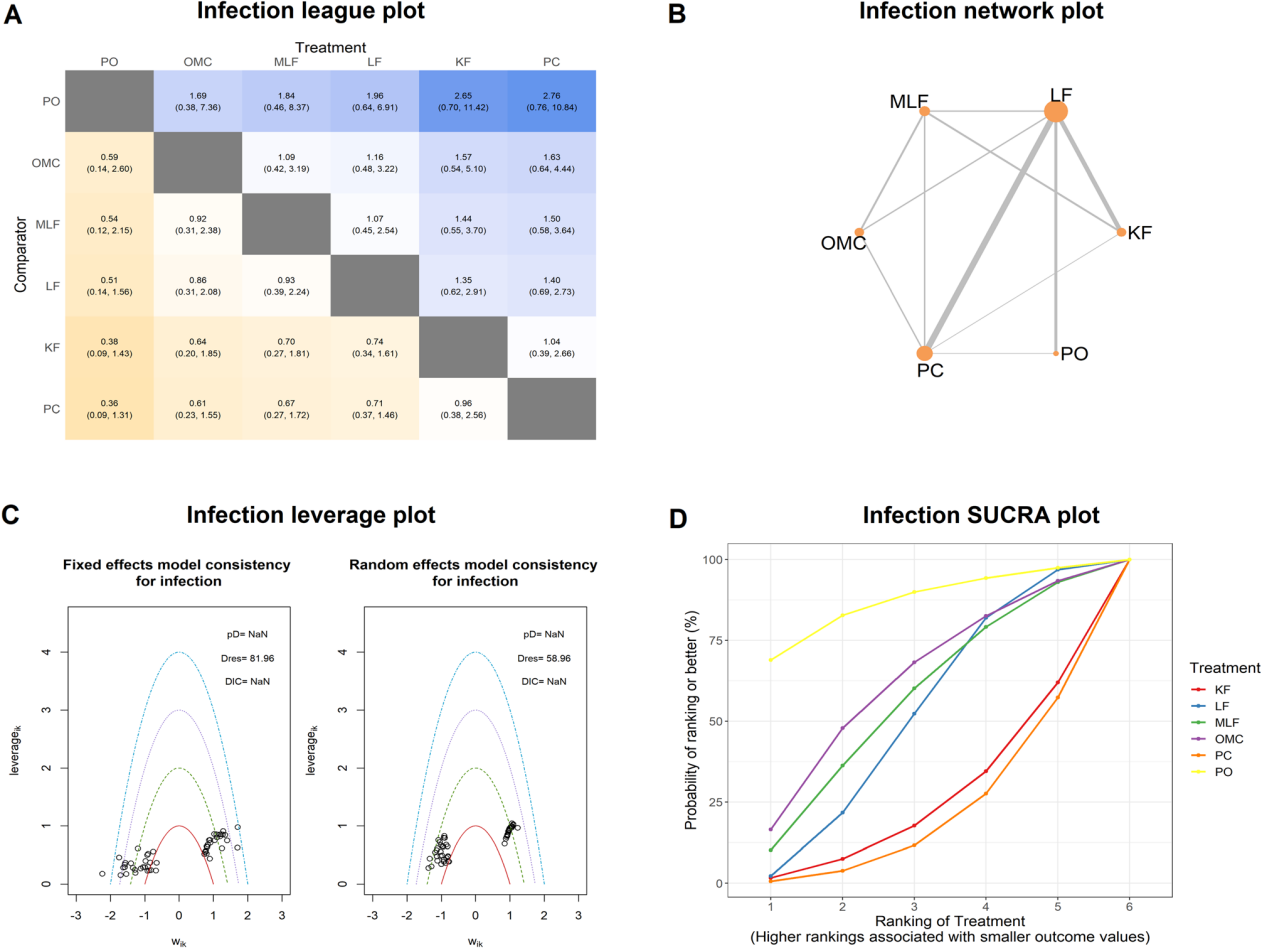
Network analysis results for infection. (**A**) The league plot for surgical interventions. The number in each cell represents the comparison between the name of column versus the name of row. Results with statistically significant are annotated with an asterisk. (**B**) The network plot showing the interventions included in the network analysis. Size of nodes represents the sample size of each intervention; edges are the frequency of comparison. (**C**) The leverage plots showing the goodness of random and fixed effects. The model with fewer outliers would be preferred. *Dres* The posterior mean of the residual deviance. *pD* The effective number of parameters, calculated as the sum of the leverages. *DIC* deviance information criterion. (**D**) The surface under the cumulative ranking curve (SUCRA) plot. *KF* Karydakis flap, *PC* primary closure, *LF* Limberg flap, *MLF* modified Limberg flap, *OMC* off-midline closure, *PO* primary open.

**Figure 7:**
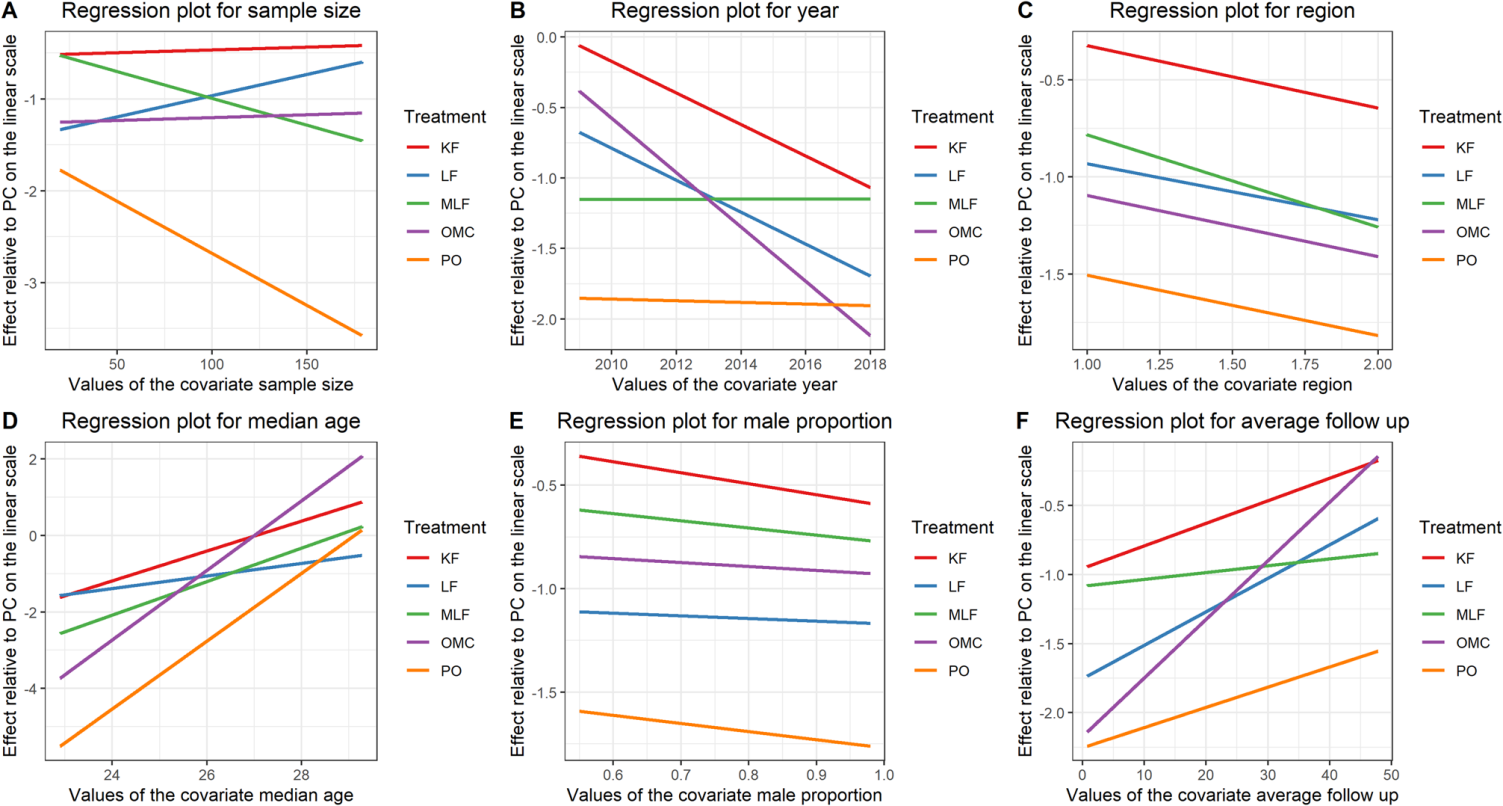
Meta-regression results for infection rate adjusted by (**A**) sample size, (**B**) year, (**C**) region, (1 for the Mediterranean area and 2 for the other regions) (**D**) median age, (**E**) male proportion, (**F**) average follow-up. All regressions were plotted as effect relative to primary closure (PC) on the linear scale.

The pooled results of 8 studies showed a significant increase in the infection rate in PC compared with LF in pairwise meta-analysis. (RR = 2.94, 95% CI 1.59, 5.44, Table 1) The authors excluded one heterogeneous comparison and 5 single study comparisons. 26 studies with 6 interventions were selected for network meta-analysis with 3,383 patients. Node splitting results showed an overall consistency profile except OMC versus MLF and PC versus OMC (Table 1). The detailed results were presented in Fig. 6, primary open (PO) has ranked the best approach with the least infection rate followed by the off-midline closures. (LF, MLF, OMC and KF) The authors illustrated meta-regression results in Fig. 7. Notably, although PC was ranked the worst approach, with the increase of median age, the incidence of infection would converge for all the other approaches to a similar level of PC (Fig. 7D). The OMC also showed a significant increase when adjusted for average follow-up duration (Fig. 7F).

### Other results

For the other complications, KF versus MLF and KF versus LF showed a significant increase in wound dehiscence and seroma in pairwise-meta results, respectively. (RR = 3.36, 95% CI 1.6, 7.05 and RR = 2.31, 95% CI 1.21, 4.43) After the selection process, only seroma and wound dehiscence with 1,257 patients and 2,995 patients were included in network meta-analysis with 4 and 5 interventions respectively. There was little inconsistency in seroma but 3 comparisons needed to be concerned in wound dehiscence. (see Supplementary Figs. S8, S9) LF was associated with the highest wound dehiscence rate but a relatively low seroma rate. On the other hand, MLF could lower the occurrence of these two complications.

For other results, we chose the day as the unit for healing time, hospitalization period and minute for operation time. We used the pain after surgery assessment time point of day-1 post-surgery to achieve a consistent result. The time differences between the interventions were not clinically significant in pairwise meta-analysis. (see Supplementary Table S3) The network meta-analysis for these two outcomes had 1,087 and 925 sample sizes for hospitalization time and for operation time. KF outperformed the other three approaches by several minutes less in operation time (see Supplementary Figs. S10, S11). The detailed regression results were summarized in Supplementary Figs. S12–S15. Especially, when compared with PC, the operation time positively correlates with the year and sample size and negatively correlates with median age and average follow-up time.

## Discussion

The recurrence rate of PSD has been a heated research topic that varies greatly in different factors including surgical interventions^54^ and geography^55^. Patients’ satisfaction and life quality after surgery have been seriously influenced by the re-emergence of PSD. Stauffer^56^ et al. conducted a thorough systematic review and meta-analysis with more than 80,000 PSD patients over the past 180 years. They concluded that the recurrence rate of PSD is highly dependent on follow-up time and the primary midline closure was associated with the highest rate of recurrence. These findings were consistent in our results. In another^57^ meta-analysis focusing on PSD children, the researchers asserted the results were generally worse than those reported in PSD adults. The lower recurrence rate of OMC and MIT showed in the current study was noticed in their results as well. Moreover, in the present study, the recurrence rate decreased from the Mediterranean area to the other areas such as Pakistan, Switzerland, Netherlands, etc. Combining the results together, the rate of recurrence could associate with age in a complicated manner which is worth investigating in the future. On the other hand, the follow-up duration could be a key factor to monitor the recurrence rate. As previous meta-analyses^56^ demonstrated, the long-term recurrence rate for primary midline closure reached 67.9% at 240 months, which soars from the 7% and 16.8% at 24 months and 60 months respectively. Regarding other approaches, the advancement flaps and rotational flaps have a smaller amount of increase in the recurrence rate: from 0.2 to 1.9% and 0.4 to 5.2% at 12 to 60 months separately. About 40% of patients who underwent phenolisation approach had recurrence at 60 months while only 1.9% at 12 months. It is important for future clinical trials to be designed with longer-term follow-up (i.e., 5–10 years), for a more reliable conclusion to be drawn.

In terms of infection and other complications, the results were agreed that PC and KF were associated with a higher occurrence rate comparatively. In previous meta-analysis^1^, the significant difference in infection rate between PO and PC was also illustrated. For midline primary closure^58^, surgical site infections occur in up to 24% of patients after surgical excision. A number of approaches were taken to decrease the infection rate and enhance the prognosis including phenolisation of the sinus tract^59^, antimicrobial irrigation^60^ and hydrocolloid dressings^58^. Based on our results, the OMC and MLF could be a promising intervention as they showed relatively lower rates of infection, wound dehiscence, and seroma incidence.

The off-midline approach was firstly published by George Karydakis as he proposed a simple lateral flap advancement technique later known as Karydakis flap^61^, which keeps the scar off the midline and flattens the natal cleft. In the following years, several off-midline approaches were categorized such as advancement flap (Karydakis flap, Bascom flap), local advancement flap (V–Y advancement flap), and rotational flap (Limberg flap, modified Limberg flap^62^, gluteus maximus myocutaneous flap). In the present study, we categorized the off-midline closures into KF, LF, MLF and OMC. As a result, the inevitable heterogeneity in OMC should be minded. Moreover, modification to the existing procedures was still a hot topic for researchers. In the present study, the MLF only showed a significant reduction in wound dehiscence and hospitalization time than LF. Recently, an eyedrop-shaped, modified Limberg transposition flap was designed and asserted to reduce wound tension and increase postoperative patient comfort.

A few limitations should be noted for our study. Firstly, the inclusion of both recurrent and primary PSD patients and the inconsistency of follow-up duration would add heterogeneity to the study. Secondly, the authors did not show the correlation between the time and recurrence or other complications in a cumulatively way. Additionally, a linear regression model was fitted to all the regressions analyzed which might not be the best way to analyze the correlation for the data. Nonetheless, to our knowledge, the present study is the first study using the network meta-analysis method to compare different surgical interventions with RCT studies. Moreover, we achieved good consistency in our study through strict screening studies for network meta-analysis. The potential relationship between each outcome and factors including region, publication year, median age, sample size, follow-up duration and male proportion were investigated using meta-regression analysis. There are several ongoing registered reviews focusing on different aspects of PSD, including the anesthesia method (PROSPERO 2020 CRD42020159358), dressings and topical agents (PROSPERO 2020 CRD42020168876) after surgery.

In summary, we have systematically reviewed all eligible RCTs relating to pilonidal sinus surgical interventions and performed a comprehensive network meta-analysis comparing different procedures with various outcomes. Although there were some significant results for the comparison between KF and LF, the superiority is still not determined. OMC and MLF provided relatively low recurrence and complications rates and therefore, could be two promising surgical interventions for PSD patients. MIT was associated with lower complications rates at the cost of a comparatively high recurrence rate. However, which is the best choice within OMC still needs more clinical trials with enough sample size and follow-up duration.

## Materials and methods

### Search strategy

The following English databases were searched systematically by two investigators (SWB, KBS): PubMed, Embase, Web of Science and Cochrane Library for clinical trials published from January 1, 2009, to December 12, 2019, using the following terms:(“pilonidal” OR “pilonidal disease” OR “pilonidal sinus” OR “pilonidal cyst” OR “sacrococcygeal disease” [Title/Abstract]) AND “randomized clinical trial”. To avoid the potential omission of studies, we also manually screened reference lists of previous systematic reviews and meta-analyses of pilonidal sinus. All of the titles and abstracts were screened out independently by the two reviewers (SWB, KBS) strictly following the inclusion criteria. Any differences were resolved through consensus, and if necessary, a senior reviewer (RQL) was consulted.

### Study selection and exclusion

Studies were included if they satisfied the following criteria:

1. Randomized controlled clinical trials to evaluate the outcomes for treating PSD patients using at least two modalities;
2. Patients who were diagnosed as chronic PSD with both recurrent or primary. They were randomly divided into different treatment groups;
3. The authors categorized all the treatments to fit a pre-defined classification (Supplementary files);
4. Papers reporting recurrence rate and infection rate were included as primary outcomes. We also collected the other outcomes documented in the articles as secondary outcomes. The endpoint of each outcome varies based on the longest follow-up duration of each study.

Studies were excluded by the following exclusion criteria:

1. Duplicated or abstracts without full texts or original data;
2. Enrolled-patients were reported earlier with the same primary authors;
3. Acute pilonidal sinus.

### Data extraction and quality assessment

This process was conducted independently by two reviewers (SB, KS) using a standard collection form by the Cochrane Collaboration for Systematic Reviews guidelines^63^. All disputes were consulted with the senior researchers. Information in each study was extracted including first author, publication year, region, median age, number of participants, treatments, follow-up duration, outcomes, male proportion, gender ratio (Male/Female), and primary or recurrent PSD. All the missing data were calculated following the Cochrane Collaboration for Systematic Reviews guidelines^63^.

The methodological quality of each study was evaluated based on the assessment of the following items: random sequence generation, allocation sequence concealment, blinding of participants and personnel, blinding of the outcome assessment, incomplete outcome data, selective reporting, and other biases. For each study, every item was rated as “low risk of bias,” “high risk of bias,” or “unclear risk of bias”.

For each comparison in primary outcomes, the certainty of the evidence was evaluated using the CINeMA^64^ (Confidence in Network Meta-Analysis) web application which is an adaption of the GRADE (Grading of Recommendations, Assessment, Development, and Evaluations) approach for network meta-analysis. It is based on a methodological framework that considers six domains: within-study bias, reporting bias, indirectness, imprecision, heterogeneity, and incoherence. It allows confidence in the results to be graded as high, moderate, low, and very low.

### Statistical analysis

Firstly, the transitivity assumption was evaluated by comparing the distribution of potential effect modifiers (geographical factor, publication year, sample size, average follow-up duration, mean age, and percentage male) across studies. These modifiers were also selected for meta-regression analysis furtherly.

Secondly, the pair-wise meta-analysis was used to analyze the direct comparisons. The authors examined the heterogeneity between studies by I^2^ statistic^65^. A fixed-effect model was applied for comparisons with negligible heterogeneity (I^2^ < 50%)^66^. Otherwise, the random-effect model and influence diagnosis^66^ were conducted to identify the contributor of heterogeneity^67,68^ except for the comparisons with only two studies. Influence diagnosis is based on the Leave-One-Out-method, in which we recalculate the results of our meta-analysis K − 1 times, each time leaving out one study. (K equals the number of included studies) This way, studies that influence the overall estimate of meta-analysis the most would be detected. The authors identified and excluded the one with the most significant contribution to heterogeneity and calculated the adjusted results afterward. Publication bias was examined in contour-enhanced funnel plots^69^. Then, the results were reported as the mean difference (MD) for continuous outcomes and risk ratio (RR) for dichotomous outcomes with corresponding 95% confidence intervals (CIs).

Moreover, before network meta-analysis, we selected the pair-wise comparisons of each outcome based on the following criteria:

1. Insignificant heterogeneity (I^2^ < 50% and p > 0.05), with or without adjusted by sensitivity analysis;
2. Two or more studies shared the same interventions.

For each outcome, the authors performed the analysis with fixed- and random-effect model. The goodness fit of each model was assessed through leverage plots that display the corresponding effective number of parameters, total residual deviance, and deviance information criterion (DIC). According to a visual examination of the leverage plots and comparison of the DIC values, the model with fewer outliers would be preferred. Then the network plots showed the entire network with nodes as interventions and the size of nodes representing the sample size. The edges represent the number of studies. The authors used the Markov Chain Monte Carlo algorithm for every eligible outcome and based on 100,000 simulation iterations and 20,000 adaptation iterations. A thinning interval of 10 was applied, which collected 1 sample every 10 iterations. We evaluated consistency statistically using node-splitting analysis^70^ that illustrates the inconsistency between indirect and direct comparisons. The residual heterogeneity and inconsistency were also explored by several meta-regressions based on modifiers mentioned earlier. The final results were shown in league plot and treatments were ranked using the surface under the curve cumulative ranking probabilities (SUCRA^71^). All the analyses and illustrations were done in R 3.6.2 using packages: “gemtc”^72^, “rjags”^73^, “dmetar”^74^, “ggplot2”^75^, “BUGSnet”^76^ and “netmeta”^77^.

## Supplementary information

Supplementary information.

## Data Availability

Some or all data, models, or code generated or used during the study are available from the corresponding author by request.
