# Supplementary material for: Surgical procedures in the pilonidal sinus disease: a systematic review and network meta-analysis
Source: Sci Rep. 2020 Aug 13;10:13720. doi: 10.1038/s41598-020-70641-7 (PMC7426950; doi:10.1038/s41598-020-70641-7)
Supplement: Supplementary file 1 — Supplementary information. [file 41598_2020_70641_MOESM1_ESM.pdf]

Surgical procedures in the pilonidal sinus disease: A systematic review and network meta-analysis

Siwei Bi[1], Kaibo Sun[2], Shanshan Chen[2], Jun Gu[3*]
1 Department of Burn and Plastic Surgery, West China Hospital, Sichuan University, Chengdu, Sichuan, 610041, People's Republic of China.
2 West China School of Medicine, Sichuan University, Chengdu, Sichuan, 610041, People's Republic of China.
3 Department of Cardiovascular Surgery, West China Hospital, Sichuan University, Chengdu, Sichuan, 610041, People's Republic of China.
* Corresponding to: Jun Gu, M.D.

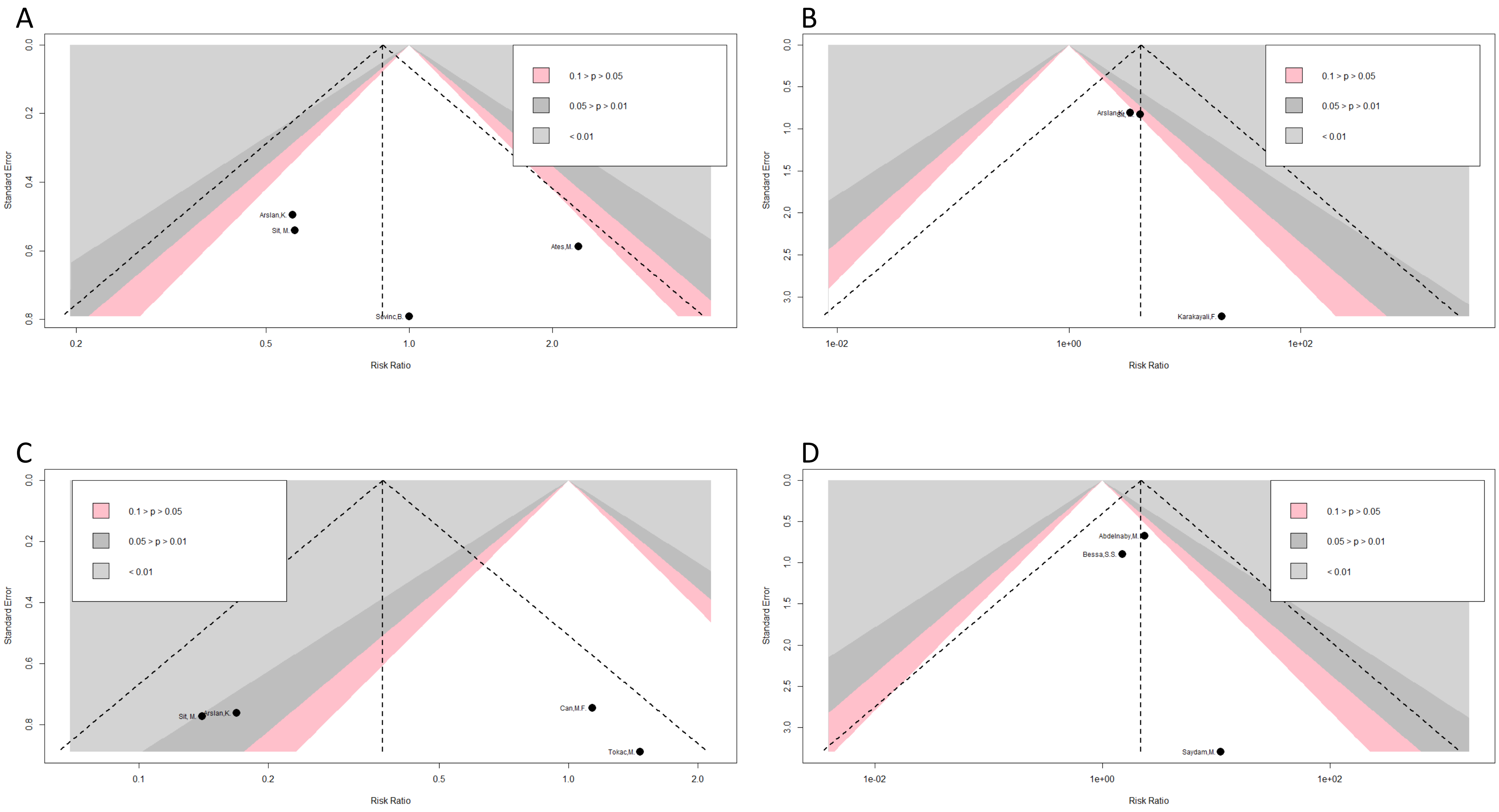

Fiugre S1. Enhanced funnel plots for recurrence rate. A. LF versus KF. B. LF versus MLF. C. MLF versus KF. D. MLF versus OMC. KF: Karydakis flap, LF: Limberg flap, MLF: modified Limberg flap.

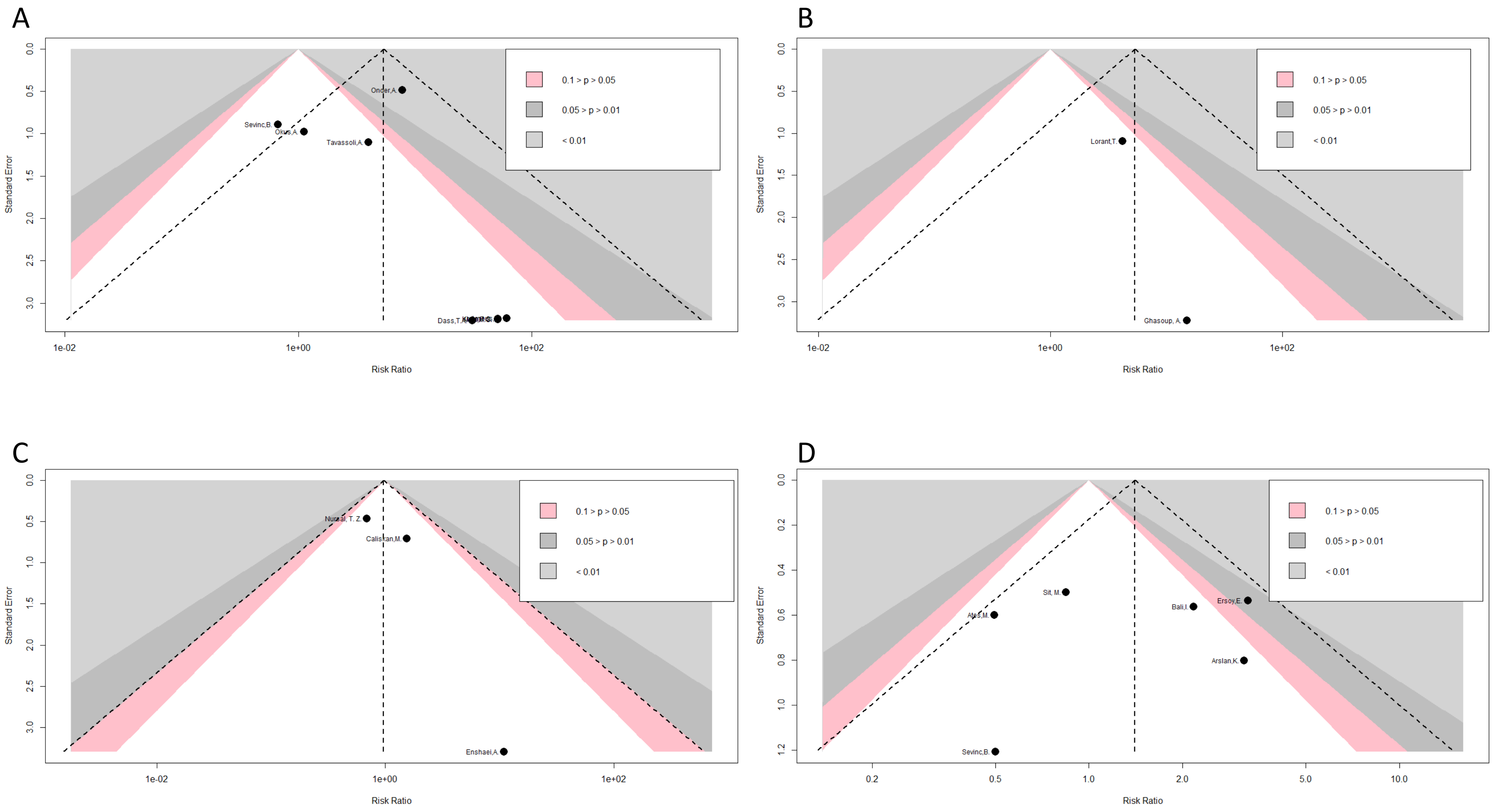

Fiugre S2. Enhanced funnel plots for recurrence rate. A. PC versus LF. B. PC versus MIT. C. PC versus OMC. D: Enhanced funnel plots for infection KF versus LF. PC: primary closure, LF: Limberg flap, MIT: Minimally invasive technique, OMC: off-midline closure, KF: Karydakis flap.

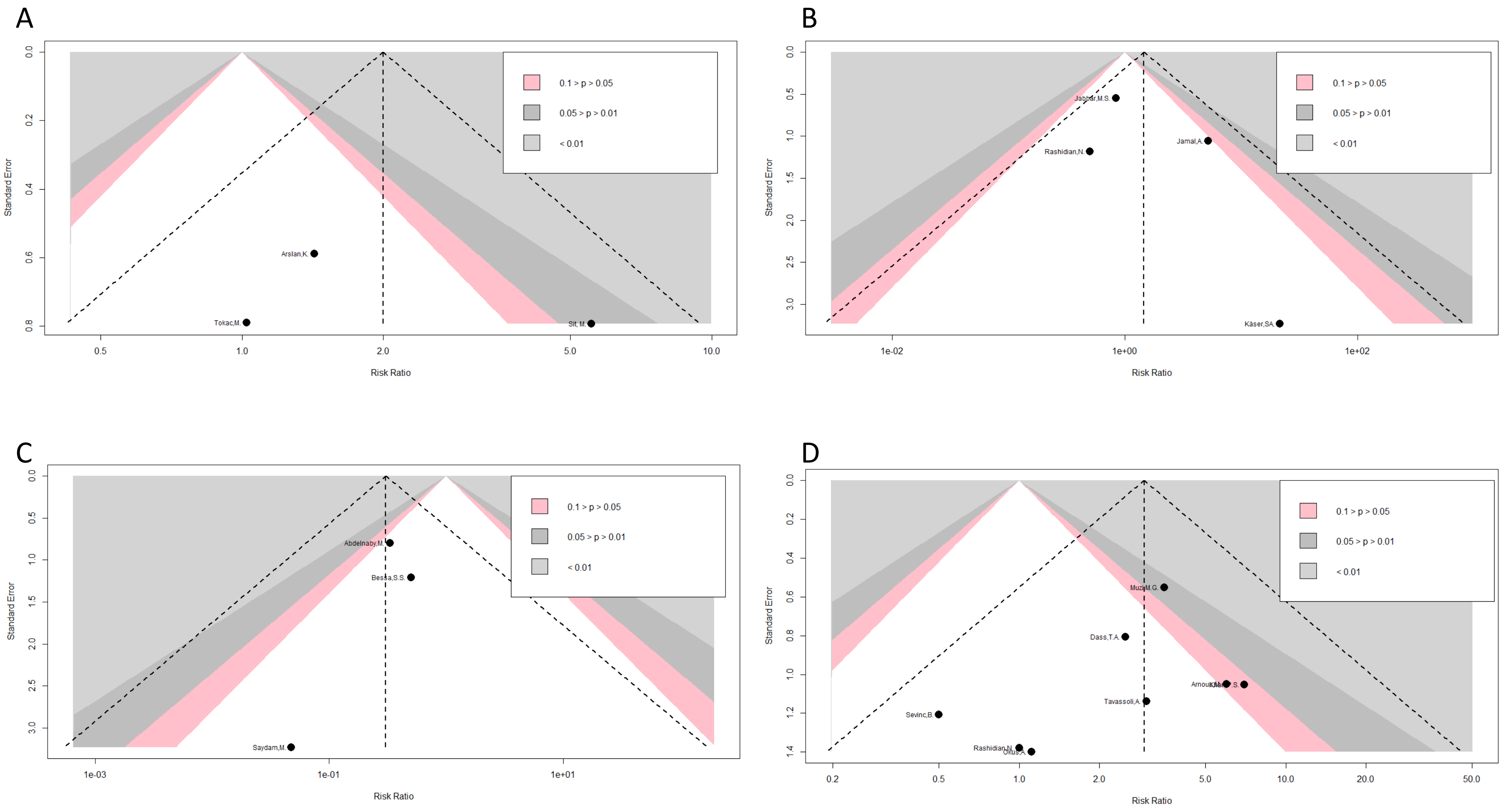

Fiugre S3. Enhanced funnel plots for infection rate. A. KF versus MLF. B. LF versus PO. C. OMC versus MLF D. PC versus LF. KF: Karydakis flap, MLF: modified Limberg flap, LF: Limberg flap, PO: primary open OMC: off-midline closure, PC: primary closure.

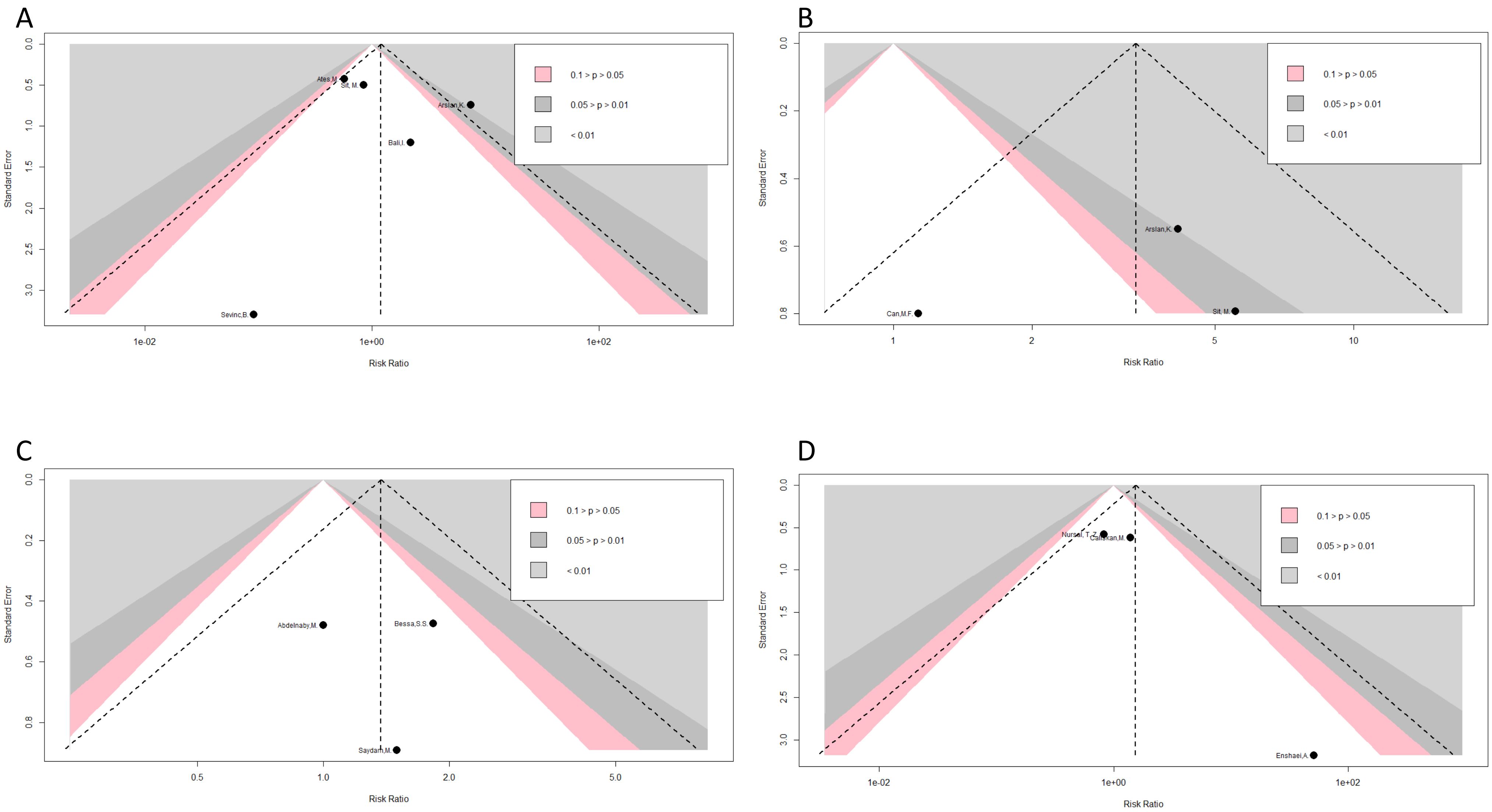

Fiugre S4. Enhanced funnel plots for wound dehiscence rate. A. KF versus LF. B. KF versus MLF. C. MLF versus OMC D. PC versus OMC. KF: Karydakis flap, LF: Limberg flap, MLF: modified Limberg flap, OMC: off-midline closure, PC: primary closure.

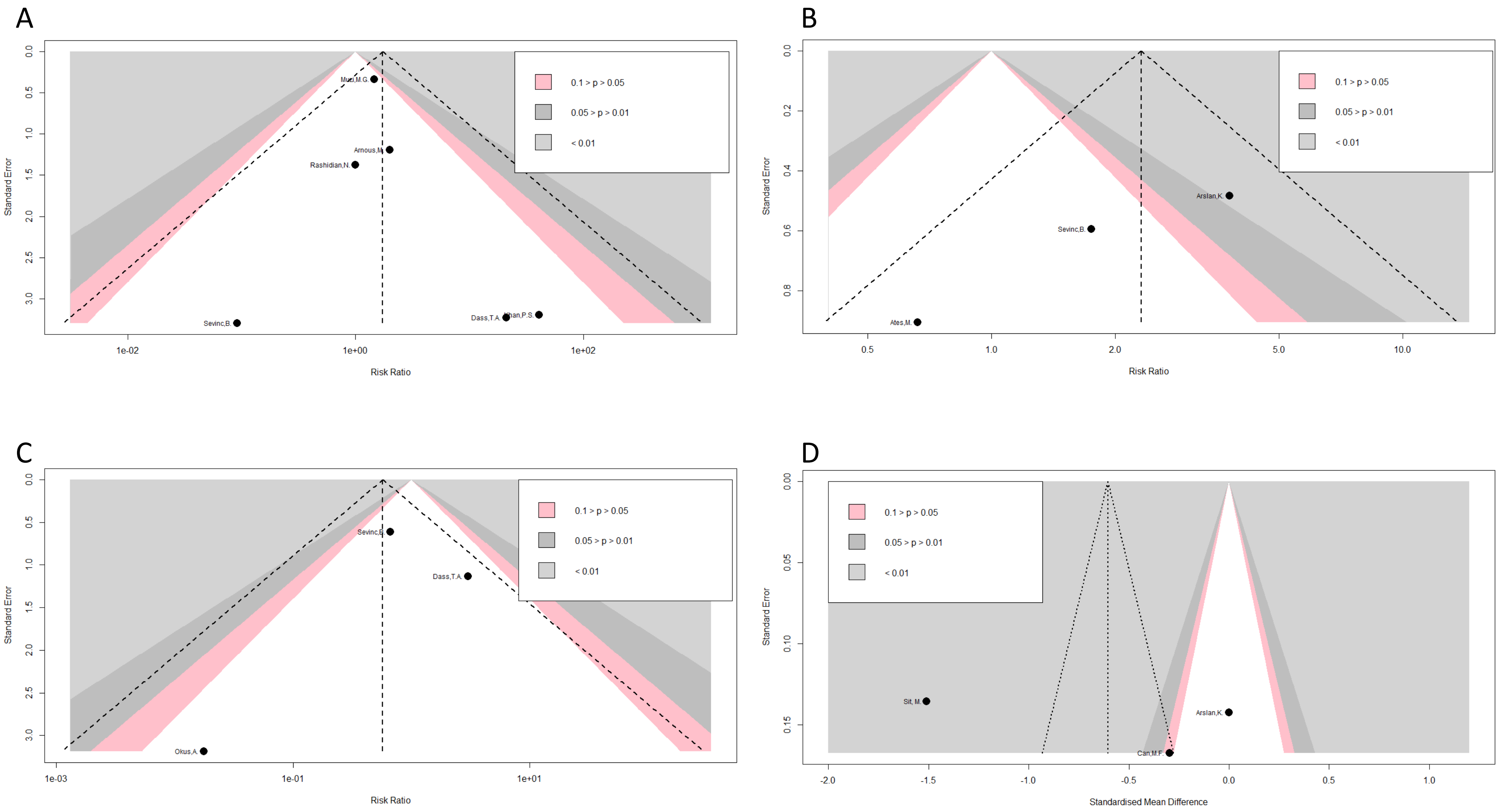

Fiugre S5. A: Enhanced funnel plots for wound dehiscence rate PC versus LF. Enhanced funnel plots for seroma rate B. KF versus LF. C. LF versus PC D: Enhanced funnel plots for hospitalization period. MLF versus KF. PC: primary closure, LF: Limberg flap, KF: Karydakis flap, MLF: modified Limberg flap.

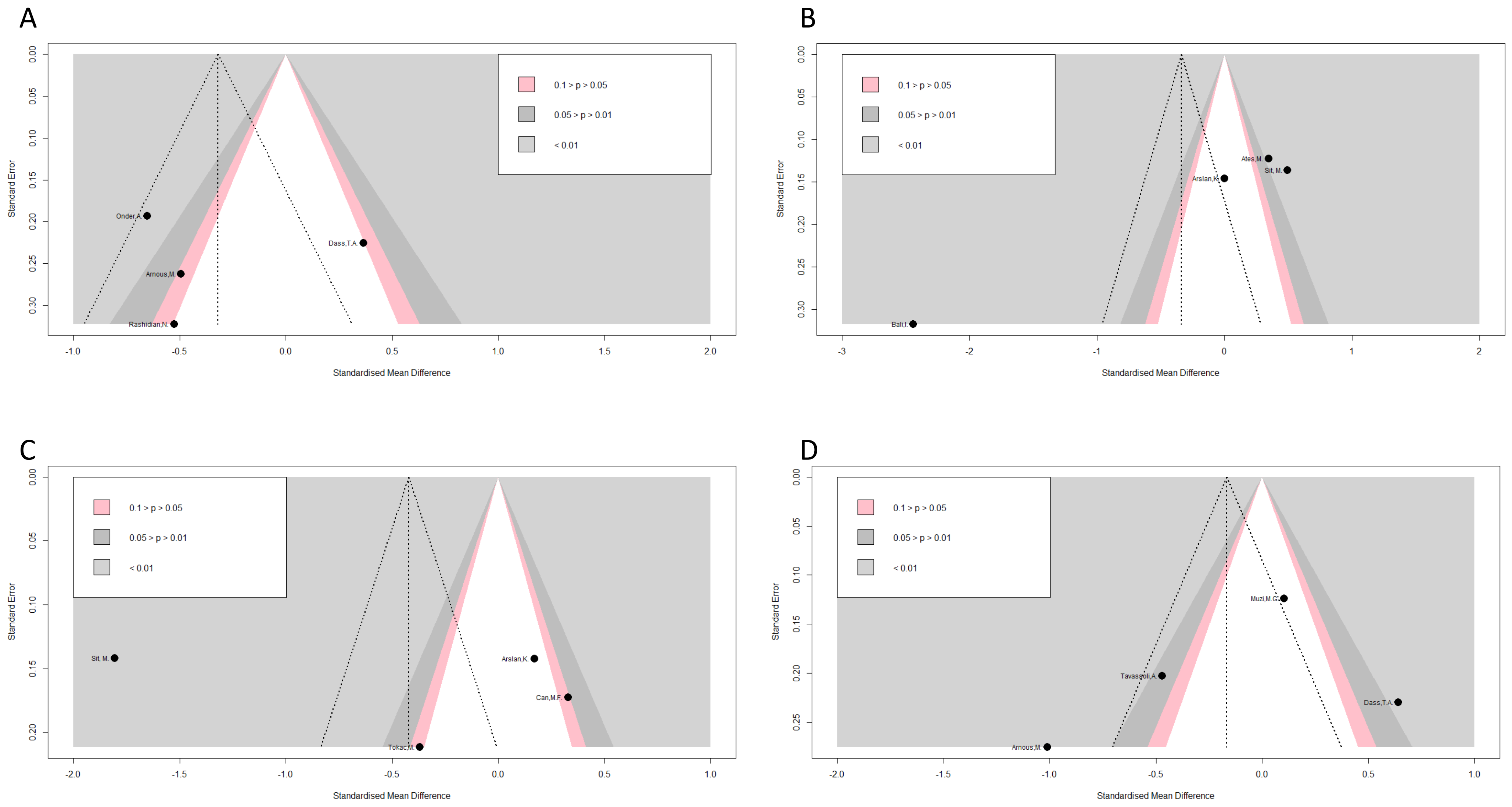

Fiugre S6. Enhanced funnel plots for hospitalization period A:PC versus LF.B: LF versus KF. Enhanced funnel plots for time off work. C. MLF versus KF D. PC versus KF. . PC: primary closure, LF: Limberg flap, KF: Karydakis flap, MLF: modified Limberg flap.

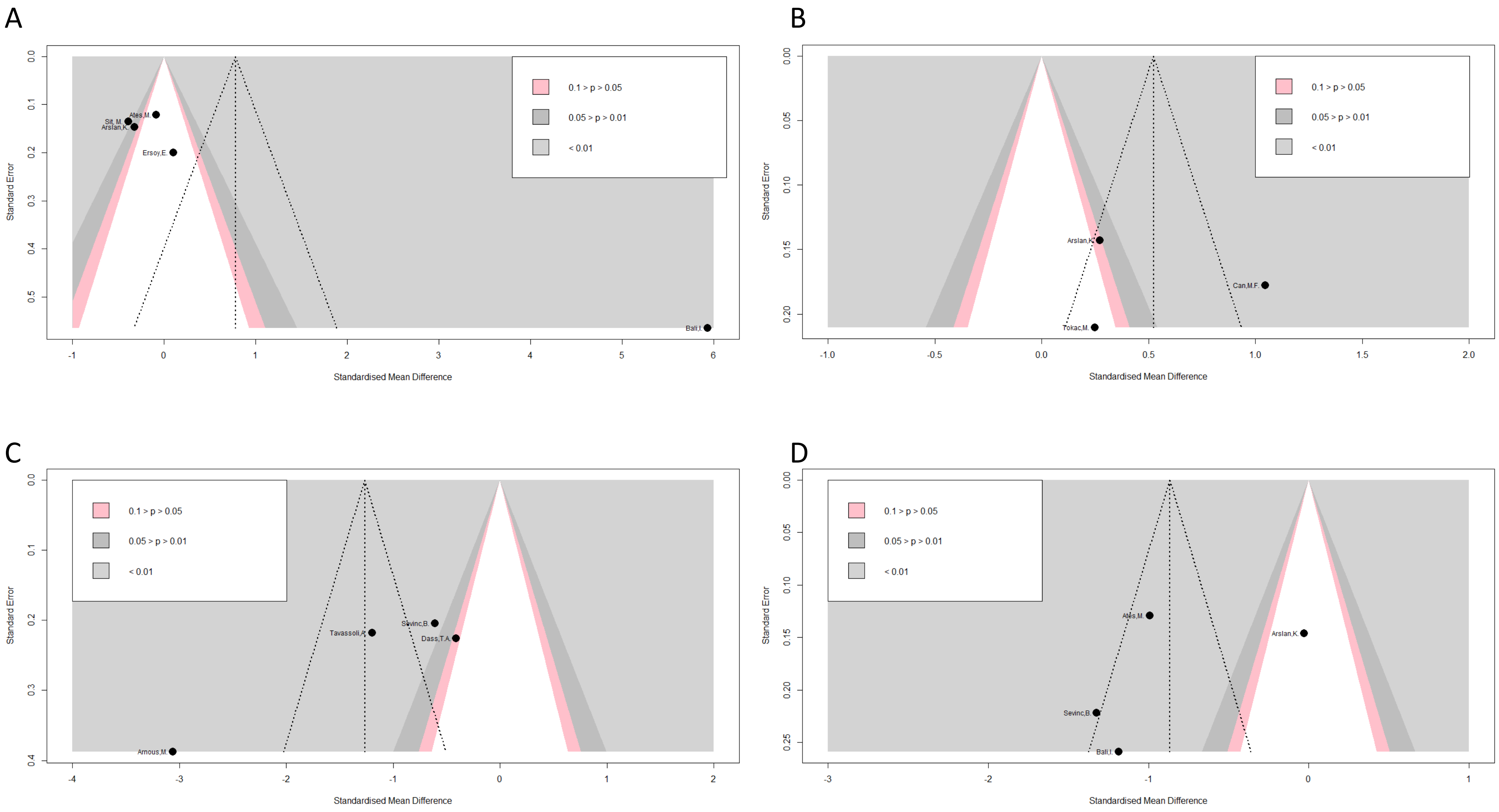

Fiugre S7. A: Enhanced funnel plots for time off work KF versus LF. Enhanced funnel plots for operation time B. MLF versus KF. C. PC versus LF D: PC versus KF. . PC: primary closure, KF: Karydakis flap, MLF: modified Limberg flap, LF: Limberg flap.

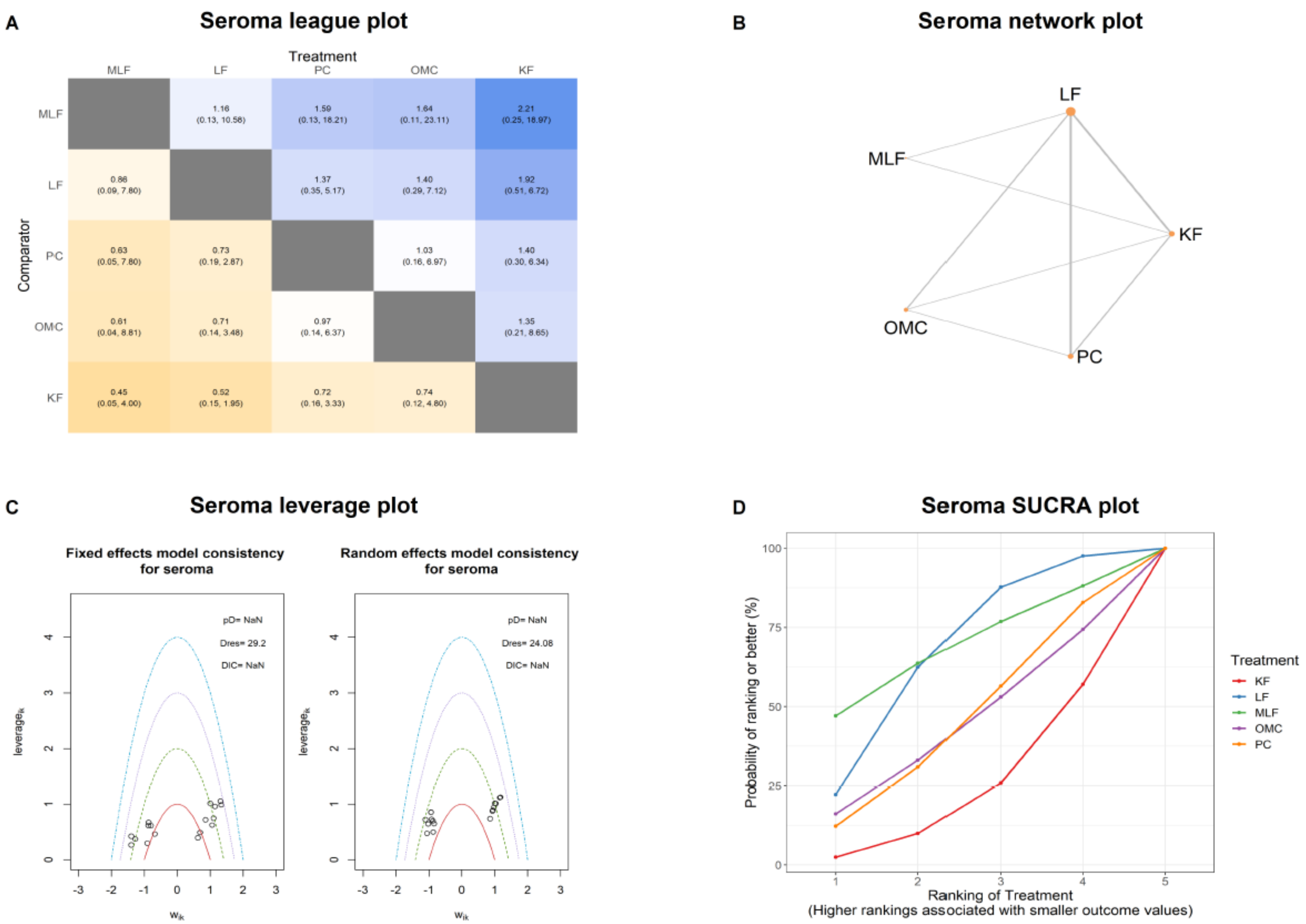

Figure S8. Network analysis results for seroma. A. The league plot for surgical interventions. The number in each cell represents the comparison between the name of column versus the name of row. Results with statistically significant are annotated with asterisk. B. The network plot showing the interventions included in the network analysis. Size of nodes represent the sample size of each intervention; edges are the frequency of comparison. C. The leverage plots showing the goodness of random and fixed effect. The model with fewer outliers would be preferred. Dres: The posterior mean of the residual deviance. pD: The effective number of parameters, calculated as the sum of the leverages. DIC: deviance information criterion. D. The surface under the cumulative ranking curve (SUCRA) plot. KF: Karydakis flap, PC: primary closure, LF: Limberg flap, MLF: modified Limberg flap, OMC: off-midline closure.

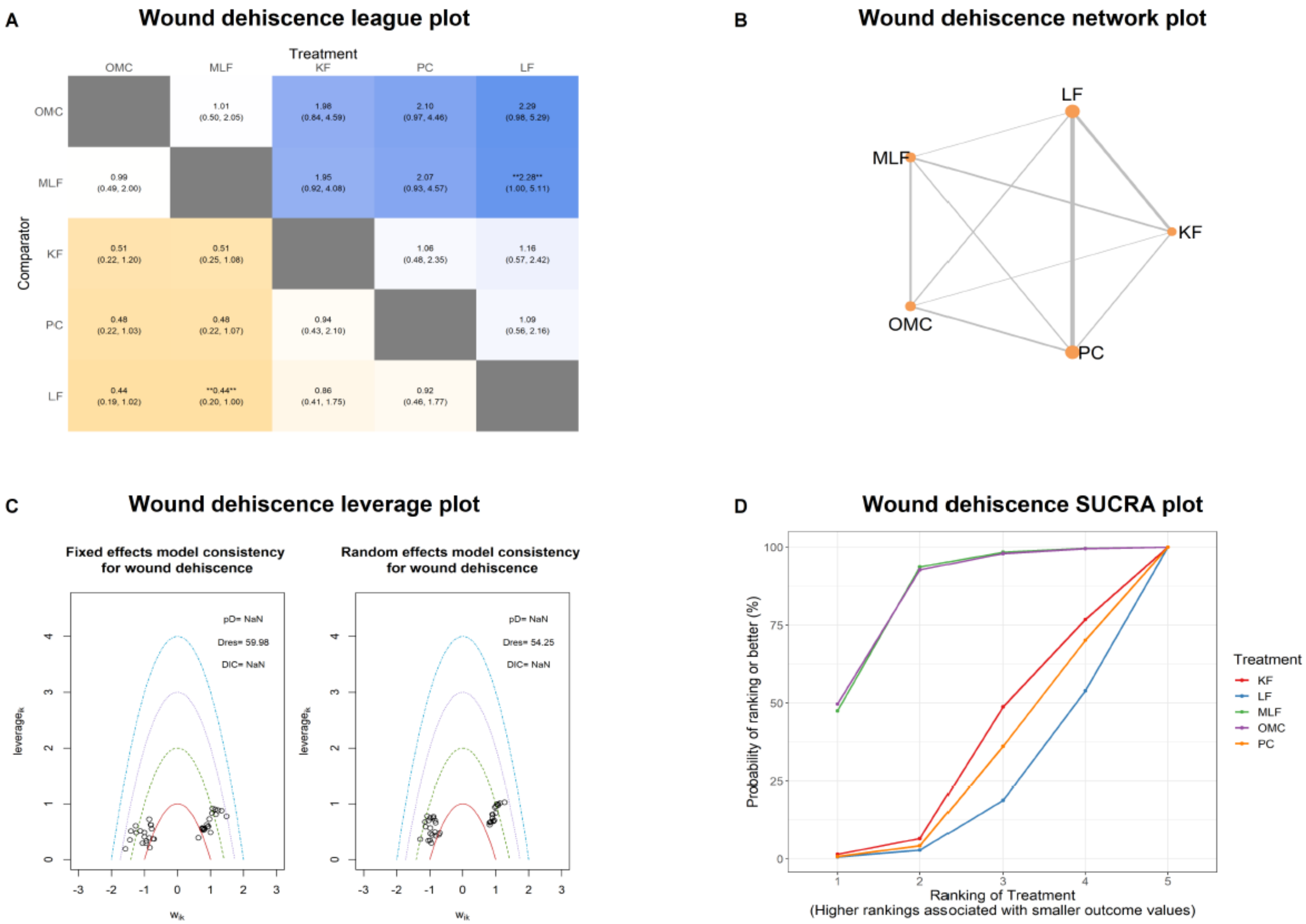

Figure S9. Network analysis results for wound dehiscence. A. The league plot for surgical interventions. The number in each cell represents the comparison between the name of column versus the name of row. Results with statistically significant are annotated with asterisk. B. The network plot showing the interventions included in the network analysis. Size of nodes represent the sample size of each intervention; edges are the frequency of comparison. C. The leverage plots showing the goodness of random and fixed effect. The model with fewer outliers would be preferred. Dres: The posterior mean of the residual deviance. pD: The effective number of parameters, calculated as the sum of the leverages. DIC: deviance information criterion. D. The surface under the cumulative ranking curve (SUCRA) plot. KF: Karydakis flap, PC: primary closure, LF: Limberg flap, MLF: modified Limberg flap, OMC: off-midline closure.

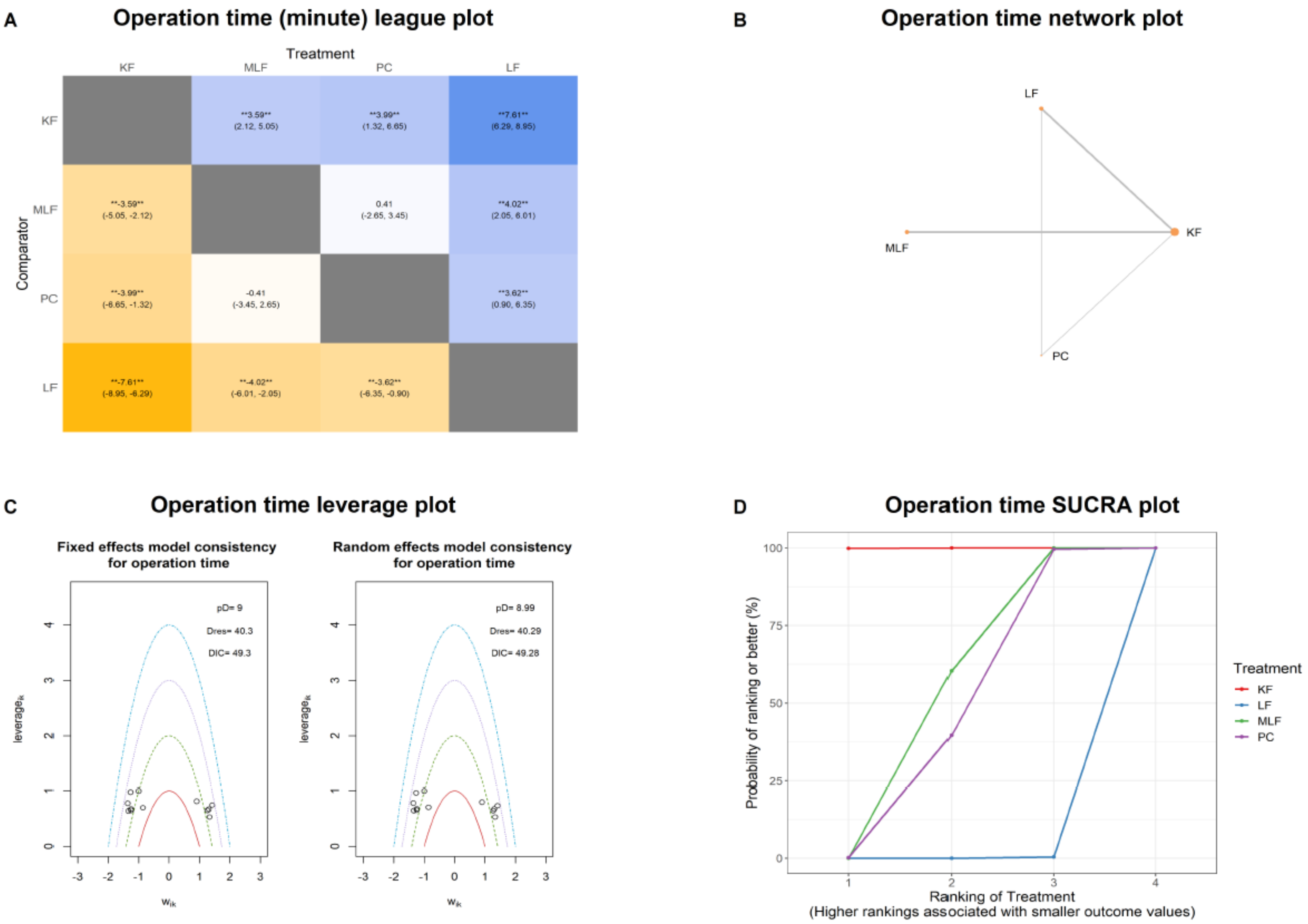

Figure S10. Network analysis results for operation time. A. The league plot for surgical interventions. The number in each cell represents the comparison between the name of column versus the name of row. Results with statistically significant are annotated with asterisk. B. The network plot showing the interventions included in the network analysis. Size of nodes represent the sample size of each intervention; edges are the frequency of comparison. C. The leverage plots showing the goodness of random and fixed effect. The model with fewer outliers would be preferred. Dres: The posterior mean of the residual deviance. pD: The effective number of parameters, calculated as the sum of the leverages. DIC: deviance information criterion. D. The surface under the cumulative ranking curve (SUCRA) plot. KF: Karydakis flap, PC: primary closure, LF: Limberg flap, MLF: modified Limberg flap.

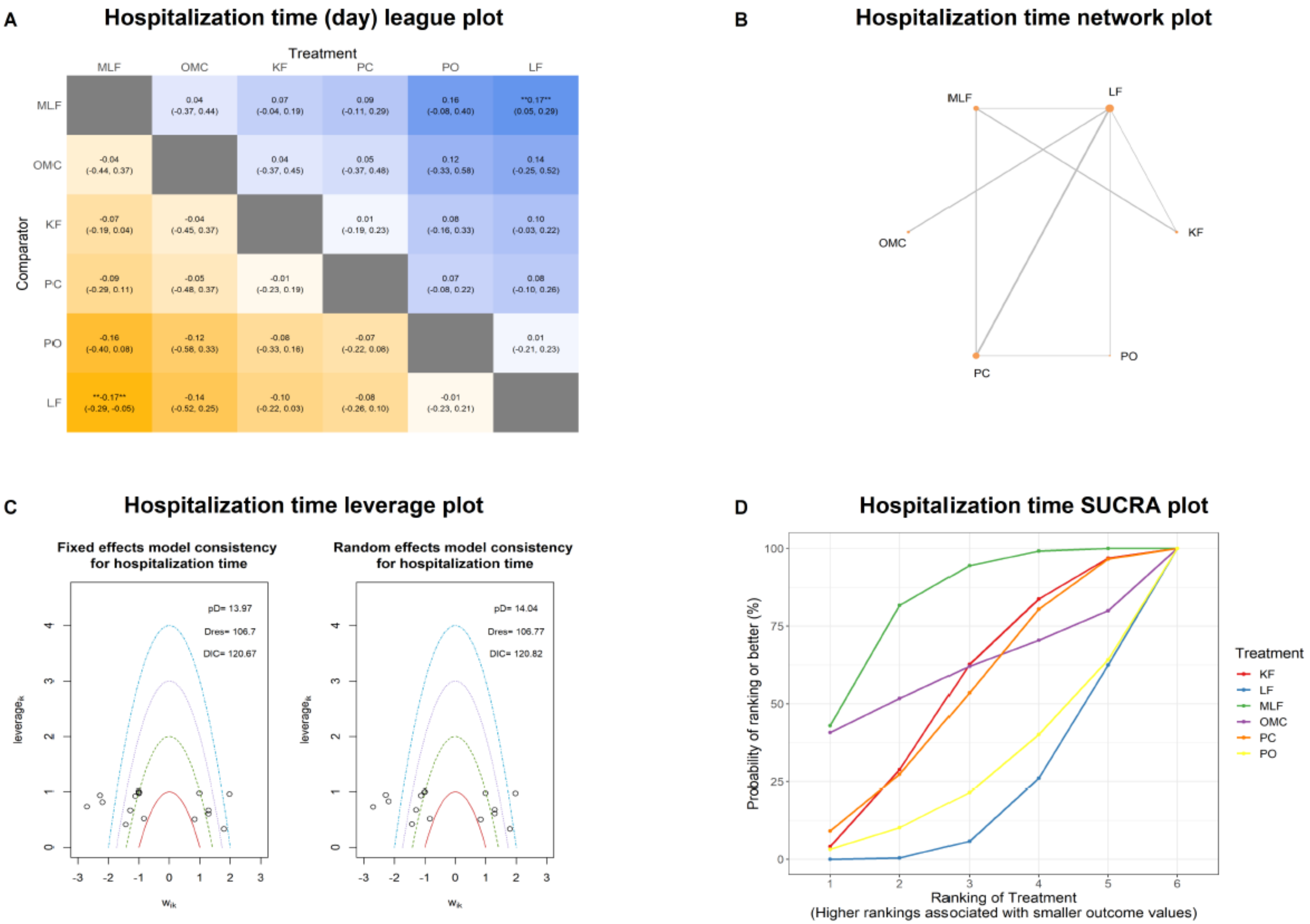

Figure S11. Network analysis results for hospitalization time. A. The league plot for surgical interventions. The number in each cell represents the comparison between the name of column versus the name of row. Results with statistically significant are annotated with asterisk. B. The network plot showing the interventions included in the network analysis. Size of nodes represent the sample size of each intervention; edges are the frequency of comparison. C. The leverage plots showing the goodness of random and fixed effect. The model with fewer outliers would be preferred. Dres: The posterior mean of the residual deviance. pD: The effective number of parameters, calculated as the sum of the leverages. DIC: deviance information criterion. D. The surface under the cumulative ranking curve (SUCRA) plot. KF: Karydakis flap, PC: primary closure, LF: Limberg flap, MLF: modified Limberg flap, OMC: off-midline closure, PO: primary open.

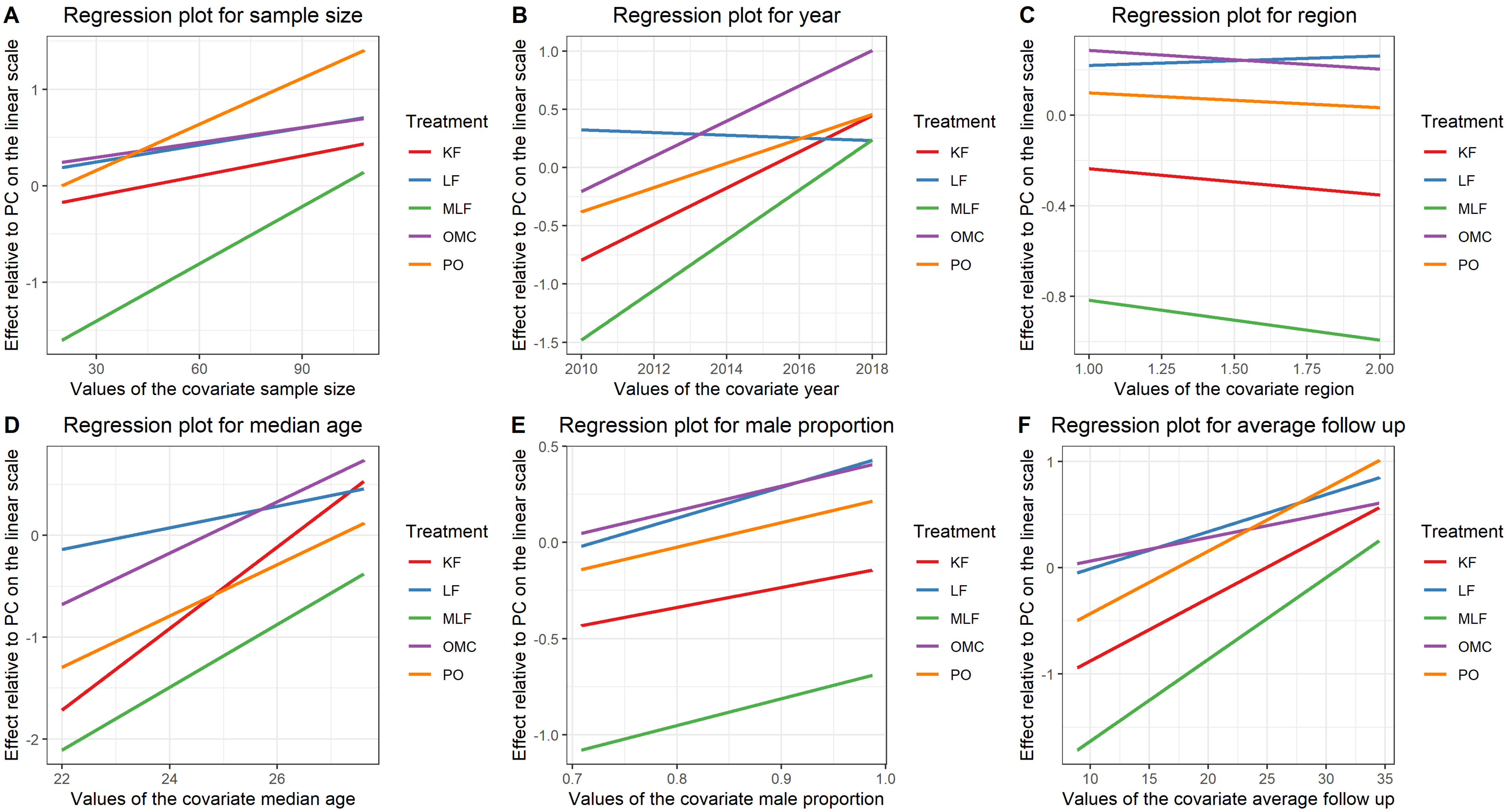

Figure S12. Meta-regression results for hospitalization time adjusted by A sample size, B year, C region, (1 for the Mediterranean area and 2 for the other regions) D median age, E male proportion, F average follow-up. All regressions were plotted as effect relative to primary closure (PC) on the linear scale.

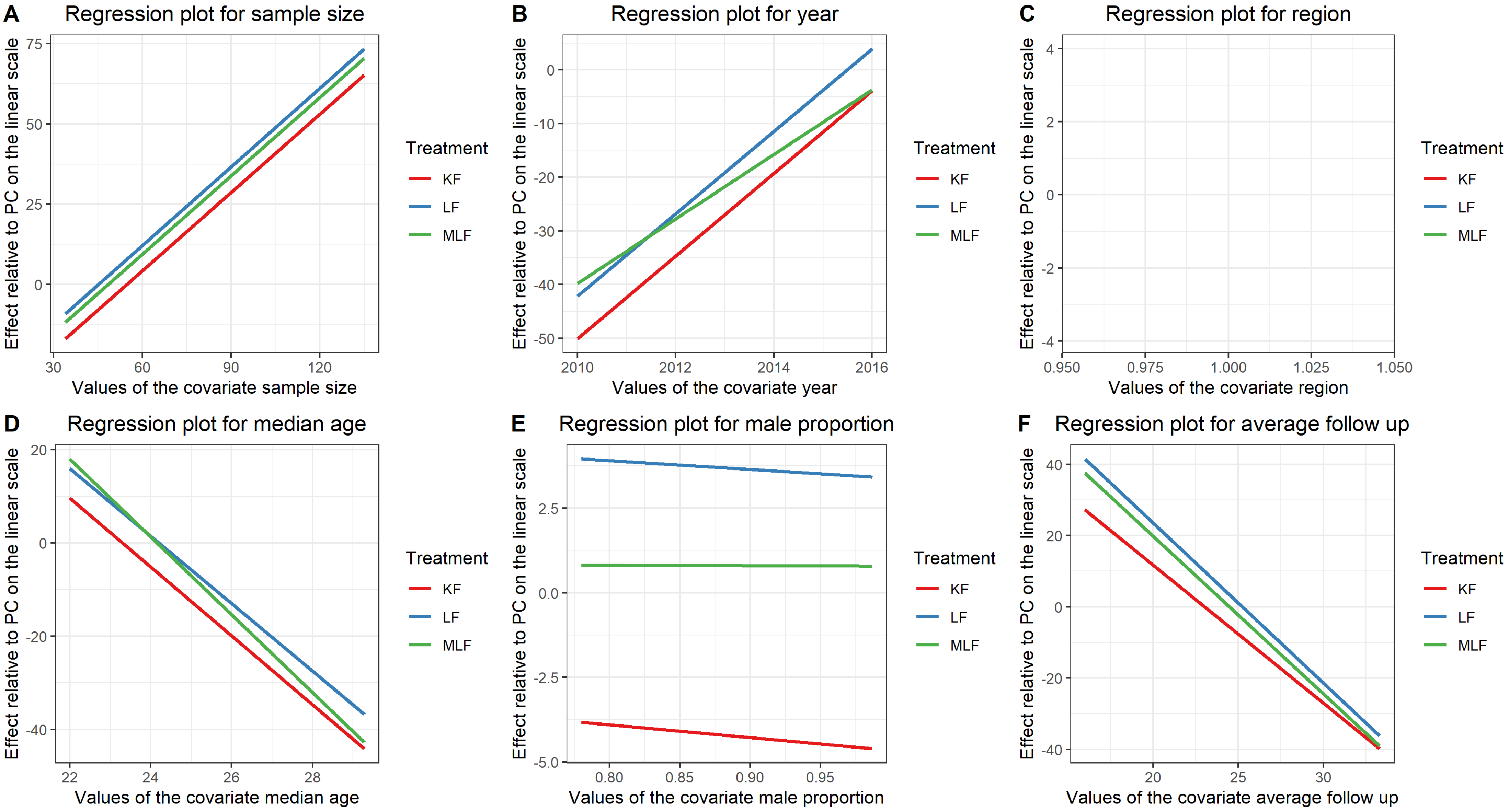

Figure S13. Meta-regression results for operation time adjusted by A sample size, B year, C region, (1 for the Mediterranean area and 2 for the other regions) D median age, E male proportion, F average follow-up. All regressions were plotted as effect relative to primary closure (PC) on the linear scale.

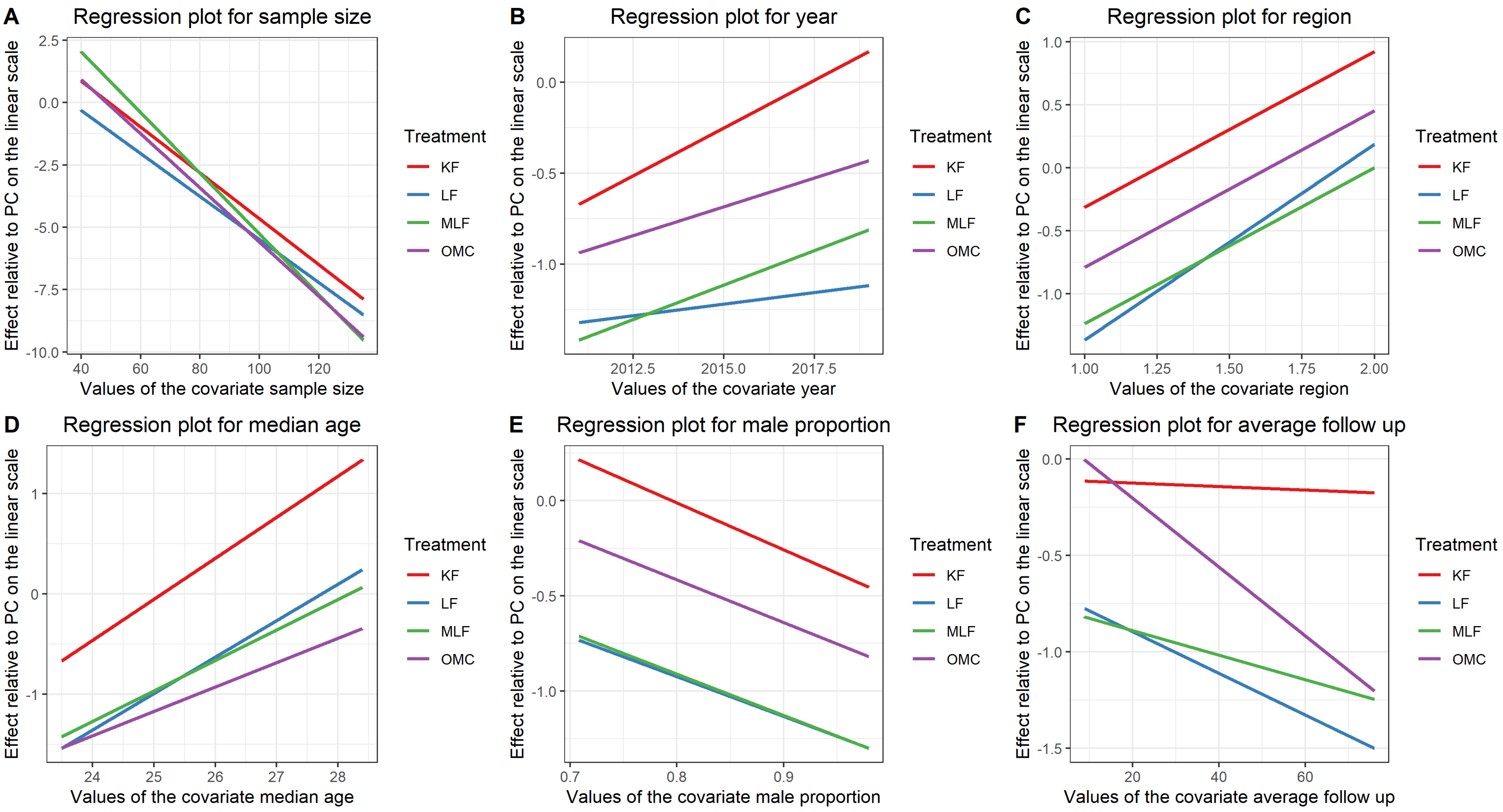

Figure S14. Meta-regression results for operation time adjusted by A sample size, B year, C region, (1 for the Mediterranean area and 2 for the other regions) D median age, E male proportion, F average follow-up. All regressions were plotted as effect relative to primary closure (PC) on the linear scale.

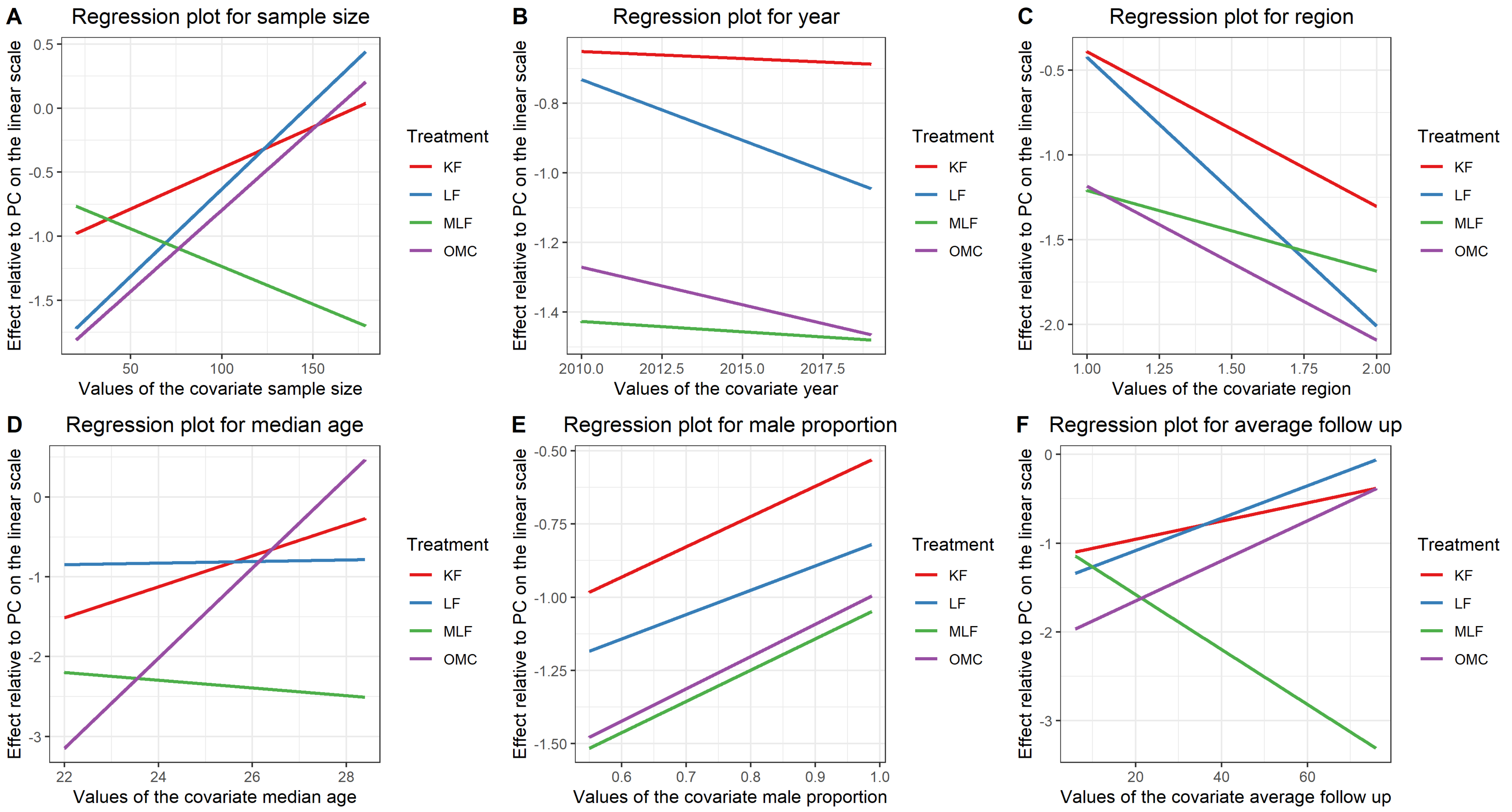

Figure S15. Meta-regression results for wound dehiscence adjusted by A sample size, B year, C region, (1 for the Mediterranean area and 2 for the other regions) D median age, E male proportion, F average follow-up. All regressions were plotted as effect relative to primary closure (PC) on the linear scale.

Table S1. Classifications of surgical interventions in the network meta-analysis.

Primary open (PO)	Open procedure, sinusectomy, excision with open healing, open excision with healing by secondary intention/secondary wound healing, lay open after resection
Primary closure (PC)	Primary closure, excision and primary closure, sinus excision and primary closure, elliptical excision with primary midline closure, midline unshifted adipofascial turn-over flap, tension-free primary closure, primary surgical repair
Karydakis flap (KF)	Karydakis flap
Limberg flap (LF)	Limberg flap, rhomboid flap, excision and Limberg flap, rhomboid excision followed by Limberg flap
Modified Limberg flap (MLF)	Modified Limberg flap, modified Limberg flap transposition, modified Limberg flap procedure with excision
off-midline closure (OMC)	Bascom cleft lift/closure, radical excision with off-midline closure, midline shifted adipofascial turn-over flap, rotation flap, rotational gluteal flap, Mutaf technique, lateral advancement flap transposition with Burow's triangle, radical excision with off-midline closure, modified Karydakis flap<br>V-Y advancement flap
Minimal invasive technique (MIT)	Lay open technique, phenol injection, sinotomy technique, Bascom's simple surgery, video assisted ablation of pilonidal sinus (VAAPS), unroofing and marsupialization, pit excision with phenolisation

Table S2.

Author	Country	Median age	Male proportion	Procedure	Sample size	Average follow up (months)	Outcome
Milone,M 2019	Italy	25.5	0.79	MIT	74	60	Recurrence rate, satisfaction outcome, cosmetic outcome and cost-effectiveness results.
	Italy	25.7	0.78	OMC	67	60	
GalalElshazly, W. 2011	Egypt	27	0.77	PC	60	21.5	Operation time, pain after surgery assessment, hospitalization period, time off work, total complication and recurrence rate
	Egypt	26	0.75	MLF	60	22	
Caliskan,M. 2019	Turkey	26.3	0.82	PC	72	76	Hospitalization period, total complication and recurrence rate
	Turkey	25.6	0.84	OMC	67	76	
	Turkey	24.8	0.81	KF	53	76	
Arnous,M. 2018	Egypt	25.1	0.83	PC	30	17.03	Recurrence rate, total complication, time off work and cosmetic outcome
	Egypt	23.8	0.90	LF	30	16.4	
Jabbar,M.S. 2018	Pakistan	28.43	0.90	PO	30	0.75	Infection rate
	Pakistan	27.4	0.97	LF	30	0.75	
Abdelnaby,M. 2018	Egypt	22.9	0.78	OMC	95	22	Healing time, recurrence rate, total complication
	Egypt	24.1	0.73	MLF	94	26	
Milone,M. 2018	Italy	26.49	0.83	MIT	40	45.33	Operation time, recurrence rate, infection rate, time off work, pain after surgery assessment, satisfaction outcome
	Italy	25.42	0.75	PO	40	44	
Calikoglu,I. 2017	Turkey	30.1	0.77	MIT	70	38	Healing time, pain after surgery assessment, time off work, recurrence rate, usage of pain medication
	Turkey	28.9	0.79	PO	70	45	
Ghasoup,A. 2017	Jordan	22	0.79	PC	28	8	Recurrence rate
	Jordan	22	0.79	MIT	20	8	
Zorlu,Musa 2016	Turkey	NA	0.80	LF	41	9.2	Recurrence rate, total complication, healing time, pain after surgery assessment
	Turkey	NA	0.80	OMC	40	8.9	
Sevinc,B. 2016	Turkey	24	0.82	KF	50	24.2	Recurrence rate, total complication
	Turkey	23.5	0.78	LF	50	24.2	
	Turkey	23.5	0.86	PC	50	24.2	

Tokac,M. 2015	Turkey	29.28	0.87	MLF	46	26.56	Hospitalization period, infection rate, time off work, drain stay time, painless sitting time, painless toilet-sitting time, and painless walking time
	Turkey	28.35	0.87	KF	45	25.46	
Saydam,M. 2015	Turkey	23	NA	MLF	50	12	Pain after surgery assessment, recurrence rate, infection rate, total complication.
	Turkey	23	NA	OMC	50	12	
Keshvari,A. 2015	Iran	26.43	0.84	KF	161	49	Healing time, time off work, total complication, recurrence rate.
	Iran	25.37	0.72	PO	160	49	
Furnee,E.J. 2015	Netherlands	NA	NA	OMC	50	12	Time off work, recurrence rate, usage of pain medication
	Netherlands	NA	NA	MIT	50	12	
Bali,I. 2015	Turkey	25	0.86	LF	37	28.2	Infection rate, edema rate, hematoma rate, wound dehiscence rate, time off work, pain after surgery assessment, healing time, painless seating, satisfaction outcome
	Turkey	23.5	0.94	KF	34	27.6	
Käser,SA. 2014	Switzerland	26	0.84	LF	51	12	Time off work, pain after surgery assessment, total complication recurrence rate, satisfaction outcome
	Switzerland	24	0.78	PO	51	12	
Shabbir,F. 2014	Pakistan	NA	0.90	MLF	30	12	Infection rate, recurrence rate, hospitalization period, time off work
	Pakistan	NA	0.93	PC	30	12	
Rashidian,N. 2014	Iran	27.61	0.78	LF	20	18	Hospitalization period, healing time, time off work, recurrence rate, total complication
	Iran	27.61	0.78	PC	20	18	
	Iran	27.61	0.78	PO	20	18	
Enshaei,A. 2014	Iran	24.17	0.55	PC	40	6	Hospitalization period, pain after surgery assessment, hematoma rate
	Iran	24.17	0.73	OMC	40	6	
Emir,S. 2014	Turkey	26.5	0.55	PC	40	12	Time off work, healing time
	Turkey	25.2	0.60	MIT	40	12	
Arslan,K. 2014	Turkey	26.5	0.71	LF	96	34.5	Seroma rate, wound dehiscence rate, recurrence rate, seroma rate, hematoma rate, infection rate
	Turkey	24.7	0.88	MLF	108	32.9	

	Turkey	24.7	0.85	KF	91	33.3	
Bessa,S.S. 2013	Egypt	23	0.90	OMC	60	20.5	Operative time, total complication, cosmetic outcome, recurrence rate
	Egypt	23	0.97	MLF	60	20.5	
Sit,M. 2013	Turkey	23.9	NA	KF	113	24.7	Time off work, drain stay time, painless sitting time, painless toilet-sitting time, and painless walking time, maceration rate, recurrence rate, and hypoesthesia rate
	Turkey	25.5	NA	LF	109	26.771	
	Turkey	24.9	NA	MLF	179	28.039	
Khan,P.S. 2013	India	26	0.85	PC	60	24	hospitalization period, total complication
	India	24	0.88	LF	60	24	
Guner,A. 2013	Turkey	25.41	0.79	LF	61	13	Pain after surgery assessment, healing time, hospital stay, total complication, and early recurrence rate, excised tissue weight, quality of life scores,
	Turkey	24.64	0.82	OMC	61	13	
Dass,T.A. 2012	India	28.4	0.90	LF	40	36	Total complication, pain after surgery assessment, time off work, hospitalization period, recurrence rate
	India	28.4	0.95	PC	40	36	
Onder,A. 2012	Turkey	25.11	0.95	PC	38	27.5	Total complication and recurrence rate,
	Turkey	27.55	0.94	LF	106	27.5	
Okus,A. 2011	Turkey	24	0.98	LF	49	31.5	Recurrence rate
	Turkey	25.5	0.91	PC	44	28	
Tavassoli,A. 2011	Iran	24	0.75	PC	50	NA	Pain after surgery assessment, hospitalization period, time off work, satisfaction outcome
	Iran	24	0.75	LF	50	NA	
Lorant,T. 2011	Sweden	27	0.72	PC	39	12	Recurrence rate, infection rate
	Sweden	28.5	0.80	MIT	41	12	
Ates,M. 2011	Turkey	24.45	0.91	KF	135	26.22	Operative time, total complication, pain after surgery assessment, cosmetic outcome, hospitalization period, recurrence rate
	Turkey	25.5	0.87	LF	134	26.58	
Nursal,T.Z. 2010	Turkey	27.5	0.857	OMC	77	29.7	Recurrence rate, infection rate
	Turkey	26.1	0.744	PC	78	29.7	
Muzi,M.G. 2010	Italy	25.01	0.84	PC	130	47.83	Pain after surgery assessment, hospitalization period, time off work, total complications, recurrence rate
	Italy	25.05	0.88	LF	130	45.76	
Can,M.F. 2010	Turkey	22	0.99	MLF	77	16	Total complication, recurrence rate, satisfaction outcome, hospitalization period

	Turkey	22	0.90	KF	68	16	
Karakayali,F. 2009	Turkey	24.4	0.86	MIT	70	3	Time off work, healing time, pain after surgery assessment, operation time
	Turkey	23.2	0.75	LF	77	3	
Nordon,I.M. 2009	UK	27	0.67	MIT	29	36	Healing time, recurrence rate
	UK	27	0.67	OMC	26	36	
Jamal,A. 2009	Pakistan	26.84	0.96	PO	25	18	Operation time, pain after surgery assessment, hospitalization period, total complications, recurrence rate
	Pakistan	26.04	0.88	LF	24	18	
Ersoy,E. 2009	Turkey	27.8	0.72	KF	50	1	Total complication, time off work, pain, infection rate
	Turkey	25.8	0.64	LF	50	1	

Table S2. Basic information for all included studies. NA: not available; KF: Karydakis flap, PC: primary closure, LF: Limberg flap, MLF: modified Limberg flap, OMC: off-midline closure, PO: primary open; MIT: minimum invasive technique.

Table S3

Outcome	Comparison	Sample size	Number of studies	Omitted Study	Pair-wise meta-analysis				Network meta-analysis			Node splitting (p-Value)
					RR/SMD	95%CI		I^2	RR/SMD	95%CI		
Wound dehiscence	PO vs. LF	660	6	-	1.74	0.97	3.1	0	-	-	-	-
	PC vs. KF	225	2	-	-	-	-	-	1.06	0.48	2.35	0.95825
	PC vs. LF	660	6	-	1.74	0.97	3.1	0	0.92	0.46	1.77	0.2173
	PC vs. MLF	160	2	-	2.5	0.5	12.54	0	2.07	0.93	4.57	0.54845
	PC vs. OMC	374	3	-	1.54	0.72	3.31	0	2.1	0.97	4.46	0.1772
	KF vs. LF	849	5	Arslan,K.	1.2	0.73	1.98	0.6	0.86	0.41	1.75	0.16225
	KF vs. MLF	636	3	-	**3.36**	**1.6**	**7.05**	0.16	1.95	0.92	4.08	**0.02785**
	KF vs. OMC	120	1	-	-	-	-	-	1.98	0.84	4.59	0.4056
	LF vs. MLF	492	2	-	2.29	0.88	5.92	0	**2.28**	**1**	**5.11**	**0.05745**
	LF vs.OMC	203	2	-	0.09	0	8.66	0	2.29	0.98	5.29	0.49205
	MLF vs. OMC	410	3	-	1.38	0.75	2.53	0	1.01	0.5	2.05	0.05145
Seroma	PC vs. KF	225	2	-	0.83	0.34	2.03	0	0.72	0.16	3.33	0.9074
	PC vs. LF	273	3	-	1.74	0.72	4.24	0.3	1.37	0.35	5.17	0.7833
	PC vs. MLF	-	-	-	-	-	-	-	1.59	0.13	18.21	-
	PC vs. OMC	139	1	-	-	-	-	-	0.97	0.14	6.37	0.8454
	KF vs. LF	556	3	-	**2.31**	**1.21**	**4.43**	0.37	1.92	0.51	6.72	0.63335
	KF vs. MLF	199	1	-	-	-	-	-	2.21	0.25	18.97	-
	KF vs. OMC	120	1	-	-	-	-	-	1.35	0.21	8.65	0.4141
	LF vs. MLF	204	1	-	-	-	-	-	1.16	0.13	10.58	-
	LF vs.OMC	203	2	-	0.34	0.1	1.2	0.21	0.71	0.14	3.48	0.38665
	MLF vs. OMC	189	1	-	-	-	-	-	0.61	0.04	8.81	-

Operation time	PO vs. MIT	220	2	-	**0.56**	**0.23**	**0.88**	0	-	-	-	-
	PC vs. KF	100	1	-	-	-	-	-	**3.99**	**1.32**	**6.65**	-
	PC vs. LF	340	4	Arnous,M.	-0.74	-1.75	0.27	0.71	**-3.62**	**-6.35**	**-0.9**	-
	PC vs. MLF	120	1	-	-	-	-	-	0.41	-2.65	3.45	-
	KF vs. LF	627	4	Arslan,K.	-1.11	-1.55	0.68	0	**-7.61**	**-8.95**	**-6.29**	-
	KF vs. MLF	-	3	Can,M.F.	**-0.26**	**-0.41**	**-0.12**	0	**-3.59**	**-5.05**	**-2.12**	-
	LF vs. MLF	204	1	-	-	-	-	-	**4.02**	**2.05**	**6.01**	-
	LF vs.OMC	203	2	-	**0.76**	**0.47**	**1.05**	0	-	-	-	-
	MLF vs. OMC	220	2	-	**1.25**	**0.89**	**1.61**	0	-	-	-	-
Hospitalization (day)	PO vs. PC	40	1	-	-	-	-	-	0.07	-0.08	0.22	-
	PO vs. KF	-	-	-	-	-	-	-	0.08	-0.16	0.33	-
	PO vs. LF	89	2	-	0.42	-0.03	0.86	0.94	-0.01	-0.23	0.21	-
	PO vs. MLF	-	-	-	-	-	-	-	0.16	-0.08	0.4	-
	PO vs. OMC	-	-	-	-	-	-	-	0.12	-0.33	0.58	-
	PC vs. KF	-	-	-	-	-	-	-	0.01	-0.19	0.23	-
	PC vs. LF	344	4	Dass,T.A.	**-0.58**	**-0.86**	**-0.31**	0	-0.08	-0.26	0.1	**0.0341**
	PC vs. MLF	180	2	-	**1.18**	**0.86**	**1.5**	0	0.09	-0.11	0.29	**0.0336**
	PC vs. OMC	80	1	-	-	-	-	-	0.05	-0.37	0.48	-
	KF vs. LF	749	4	Bali,I.	-0.28	-0.9	0.33	0.69	-0.1	-0.22	0.03	0.1846
	KF vs. MLF	636	3	Sit, M.	-0.14	-1.75	2.02	0.46	0.07	-0.04	0.19	-
	KF vs. OMC	-	-	-	-	-	-	-	0.04	-0.37	0.45	-
	LF vs. MLF	492	2	-	**0.59**	**0.4**	**0.77**	0.97	**0.17**	**0.05**	**0.29**	**0.0379**
	LF vs.OMC	203	2	-	0.03	-0.24	0.31	0	0.14	-0.25	0.52	-
	MLF vs. OMC	100	1	-	-	-	-	-	-0.04	-0.44	0.37	-
Pain	PO vs. MIT	220	2	-	0.81	0.53	1.1	0.95	-	-	-	-
	PC vs. LF	160	2	-	0.54	0.22	0.87	0.95	-	-	-	-

Healing time (minute)	PC vs. LF	244	2	-	**-0.44**	**-0.72**	**-0.16**	0.97	-	-	-	-
	KF vs. LF	258	2	-	0.2	-0.04	0.45	0	-	-	-	-
	KF vs. MLF	336	2	-	-0.17	-0.39	0.04	0.73	-	-	-	-
	LF vs.OMC	203	2	-	**-0.28**	**-0.56**	**0**	0.85	-	-	-	-
Time off work (minute)	PC vs. LF	500	4	Arnous,M.	0.09	-1.27	1.44	0.85	-	-	-	-
	PC vs. MLF	180	2	-	**1.4**	**1.07**	**1.73**	0.91	-	-	-	-
	KF vs. LF	849	5	Bali,I.	-0.19	-0.53	0.14	0.48	-	-	-	-
	KF vs. MLF	719	4	Sit, M.	-0.06	-0.94	0.82	0.71	-	-	-	-
	LF vs. MLF	492	2	-	**1.02**	**0.82**	**1.22**	0.99	-	-	-	-
	OMC vs. MIT	296	2	-	**1.39**	**1.06**	**1.73**	0.98	-	-	-	-

Table S3 Summary meta-analysis results for wound dehiscence, seroma, operation time, hospitalization, pain score, healing time and time off work. The statistically significant results were shown in bold font. KF: Karydakis flap, PC: primary closure, LF: Limberg flap, MLF: modified Limberg flap, OMC: off-midline closure, PO: primary open; MIT: minimum invasive technique; RR: risk ratio; SMD: standardized mean difference; CI: confidential interval.
